# *Heliotropium ramosissimum* metabolic profiling, in silico and in vitro evaluation with potent selective cytotoxicity against colorectal carcinoma

**DOI:** 10.1038/s41598-022-16552-1

**Published:** 2022-07-22

**Authors:** Marwa A. A. Fayed, Mohamed E. Abouelela, Mohamed S. Refaey

**Affiliations:** 1 Department of Pharmacognosy, Faculty of Pharmacy, University of Sadat City, Sadat, 32897 Egypt; 2 Department of Pharmacognosy, Faculty of Pharmacy, Al-Azhar University, Assiut-Branch, Assiut, 71524 Egypt

**Keywords:** Plant sciences, Chemistry

## Abstract

*Heliotropium* is a genus of the Boraginaceae family. Its members are used in many traditional and folklore medicines to treat several ailments. Despite this widespread usage, only a few evidence-based scientific studies investigated and identified its phytoconstituents. Herein, we documented the chemical profile of the *Heliotropium ramosissimum* methanolic extract using gas chromatography-mass spectrometry (GC–MS) and liquid chromatography-tandem mass spectrometry (LC–ESI–MS/MS) and assessed its antioxidant and cytotoxic effects. The methanolic extract exhibited high phenolic content (179.74 ± 0.58 µg/mL) and high flavonoid content (53.18 ± 0.60 µg/mL). The GC–MS analysis of the lipoidal matter allowed us to identify 41 compounds with high percentages of 1,2-benzenedicarboxylic acid, bis(2-methoxyethyl) ester (23.91%), and 6,10,14-trimethylpentadecan-2-one (18.74%). Thirty-two phytomolecules were tentatively identified from the methanolic extract of *H. ramosissimum* using LC–MS/MS. These compounds belonged to several phytochemical classes such as phenolic acids, alkaloids, coumarins, and flavonoids. Furthermore, we assessed the antioxidant activity of the methanolic extract by DPPH assay and oxygen radical absorbance capacity assay, which yielded IC_50_ values of 414.30 µg/mL and 170.03 ± 44.40 µM TE/equivalent, respectively. We also assessed the cytotoxicity of the methanolic extract on seven different cell lines; Colo-205, A-375, HeLa, HepG-2, H-460, and OEC showed that it selectively killed cancer cells with particularly potent cytotoxicity against Colo-205 without affecting normal cells. Further studies revealed that the extract induced apoptosis and/or necrosis on Colo-205 cell line at an IC_50_ of 18.60 µg/mL. Finally, we conducted molecular docking on the LC–ESI–MS/MS-identified compounds against colon cancer antigen 10 to find potentially cytotoxic compounds. Binding score energy analysis showed that isochlorogenic acid and orientin had the highest affinity for the colon cancer antigen 10 protein, with binding scores of (− 13.2001) and (− 13.5655) kcal/mol, respectively. These findings suggest that *Heliotropium ramosissimum* contains potent therapeutic candidates for colorectal cancer treatment.

## Introduction

Family Boraginaceae is one of the largest families that include hundreds of genera and nearly 2 thousand species. Members of this family can be found in both tropical and temperate climates, especially in the Mediterranean. *Heliotropium*, a genus that belongs to this family, contains perennial and annual herbs with scorpioid cymes in their inflorescences as well as the morphology of the greatly modified stigmatic head in the flower^1^. In various traditional and folklore medical systems, *Heliotropium* species have been used to treat several illnesses*.* Various parts of the plant, including the leaves, roots, flowers, seeds, and the whole plant, are used to treat a variety of ailments^2^. In folk medicine, *H. aegyptiacum* is used to treat snake bites and scorpion stings^3^, while *H. indicum* is reported to be used against infected gums^4^, head lice^5^, rheumatism^6^, and gonorrhea^7^. *H. strigosum* has laxative, diuretic effects^8^, as well as its activity against breast abscesses^9^. *H. crispum* is used as a cooling agent and exerted a lactagogue effect in cattle^10^. *H. steudneri* is used to stop bleeding and to prevent infection^4^, while *H. ramosissimum* is used to treat burns^4^. Gout, inflammation, menstrual dysfunction, skin disorders, noxious bites, and rheumatism have all been confirmed to be treated with *Heliotropium* species^2^.

*Heliotropium* species contain phytochemicals that are bioactive and have powerful healing properties. Several classes of organic compounds viz. flavonoids, pyrrolizidine alkaloids, terpenoids, and quinones are very plentifully present in the *Heliotropium* genus^11^. Afghanistan, Burkina Faso, Canary Islands, Cape Verde, Chad, Djibouti, Egypt, Gulf States, Iran, Iraq, Lebanon-Syria, Libya, Mauritania, Niger, Saudi Arabia, Senegal, Sinai, Sudan, Turkmenistan, and Yemen are all home to *Heliotropium ramosissimum* (Lehm.) DC^12^. Few studies concerning the isolation of phytoconstituents from this species were reported^13^, in addition to evaluation of its total phenolic content and total antioxidant capacity using different assays^14^. This scarcity of knowledge on its phytochemical and biological properties encouraged us to document its phytochemical properties, antioxidant activity, and in vitro cytotoxic activity toward various cell lines. Moreover, we explored the compounds potentially responsible for the methanolic extract’s cytotoxic activity through *in-silico* studies.

## Materials and methods

### Statement

All experiments and methods including collection of the plant were performed following relevant national, and international guidelines and legislation of the Faculty of Pharmacy, University of Sadat City, Sadat City, Egypt.

### Plant material

**Figure 1:**
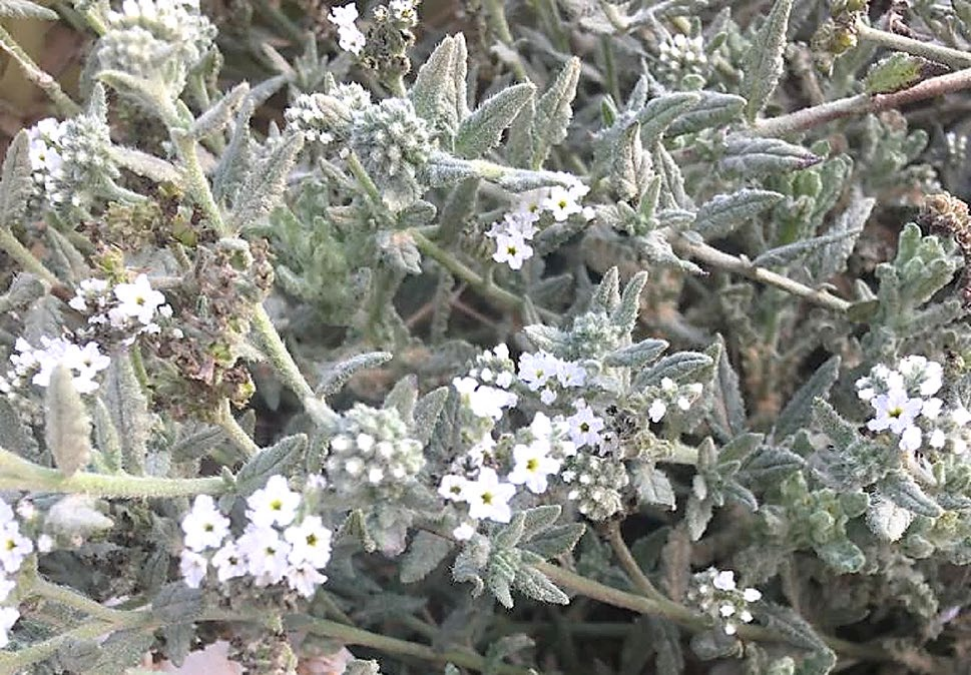
A Photo of the *H. ramosissimum* (Lehm.) DC. flowering aerial from the place of collection (taken by the corresponding author in March 2020 from El-Sadat City desert region, Sadat City, Egypt).

We collected *Heliotropium ramosissimum* (Lehm.) DC. flowering aerial parts (Fig. 1) in March 2020 from the El-Sadat City desert region, Sadat City, Egypt with license approval from the Faculty of Pharmacy, University of Sadat City, Sadat City, Egypt according to relevant guidelines and regulations. The plant material was kindly identified by Prof. Dr. A. A. Fayed, Professor of Plant Taxonomy, Faculty of Science, Assiut University, Assiut, Egypt. We deposited a voucher sample (alphabetically ordered under the letter “H”) in the Herbarium of the Faculty of Science, Assiut University, Assiut, Egypt.

### Extraction and fractionation

The air-dried powdered aerial parts of *H. ramosissimum* (Lehm.) DC. (1 kg) were defatted with *n*-hexane (3 × 1.5 L) to prepare the lipoidal matter and then extracted with methanol (3 × 1.5 L) to prepare the total methanolic extract used in this study^15^. We performed the extractions in each solvent until exhaustion. After completing the process, we removed each solvent under reduced pressure using a rotary evaporator (Acculab, USA) at 50 °C. The *n*-hexane yielded 5 g of residue, and the total methanolic extracts weighed 10 g. We stored the extracts in a vacuum desiccator until further use^16,17^.

### Phytochemical studies

#### Phytochemical screening

We identified the presence of phytochemical classes in freshly prepared crude extracts of the flowering aerial parts of *H. ramosissimum* using standard colorimetric procedures^18,19^.

#### Estimation of the total phenolic and flavonoid contents

The total phenolic content was calculated as gallic acid equivalents (GAE) per g of the sample using the Folin–Ciocalteu reagent and a calibration curve prepared with gallic acid^20^. Moreover, the total flavonoid content was determined as rutin equivalents (RE) per g of the sample using aluminum chloride (AlCl_3_) colorimetric assay^20^.

### Metabolomic analysis

#### GC–MS analysis of the lipoidal matter

The chemical composition of the lipoidal matter of the aerial parts of *H. ramosissimum* was determined using a Trace GC-TSQ mass spectrometer (Thermo Fisher Scientific, Austin, TX, USA) with a direct capillary column TG–5MS (Thermo Fisher Scientific, Austin, TX, USA) (30 m × 0.25 mm × 0.25 µm film thickness). The initial column oven temperature was 50 °C. It was then increased to 250 °C at 5 °C/min, held for 2 min, increased to the final temperature of 300 °C at 30 °C/min, and held for 2 min. The injector and MS transfer line were kept at 270 °C and 260 °C, respectively. The carrier gas (helium) had a constant flow rate of 1 mL/min. The solvent delay was 4 min, and diluted 1 µL samples were injected automatically using Autosampler AS1300 coupled with GC in split mode. We collected electron ionization mass spectra at an ionization voltage of 70 eV over the *m/z* range 50–650 in full scan mode. We set the ion source temperature to 200 °C. We identified the components by comparing their mass spectra with those of the WILEY 09 and NIST 14 mass spectral libraries^16^.

#### LC–ESI–MS/ MS profiling

We performed the LC–ESI–MS/MS analysis of the methanolic extract on an ExionLC AC system coupled with a SCIEX Triple Quad 5500 + MS/MS system equipped with an electrospray ionization (ESI) system. The samples were eluted on an Ascentis C18 Column (4.6 × 150 mm, 3 µm). Mobile phases consisted of eluent A (0.1% formic acid) and eluent B (acetonitrile, LC grade). The mobile phase gradient was programmed as follows: 10% B at 0–1 min, 10%–90% B at 1–21 min, 90% B at 21–25 min, and 10% at 25.01–28 min. The flow rate was 0.5 mL/min, and the injection volume was 10 µL. MS/MS analysis used positive and negative ionization modes with a scan (EMS-IDA-EPI) from 100 to 1000 Da for MS1 with the following parameters: curtain gas, 25 psi; Ion Spray voltage, 5500 and − 4500 v for positive and negative modes, respectively; source temperature, 500 °C; ion source gas 1 & 2, 45 psi and from 50 to 800 Da for MS2; declustering potential, 80; collision energy, 35 and − 35 for positive and negative modes, respectively; and collision energy spread, 20^21^. We identified the compounds using MS-DIAL software version 4.70.

#### Free radical scavenging activity assessment by DPPH assay

We prepared 1000 and 100 μg/mL solutions from the methanolic extract to identify a range within which the inhibitory concentration 50 (IC_50_) lay. We serially diluted the solutions exceeding 50% five times. We prepared a 100 μM Trolox stock solution in methanol. From this stock solution, we prepared 50, 40, 30, 20, 15, 10, and 5 μM solutions. We performed the 2,2-diphenyl-1-picryl-hydrazyl-hydrate (DPPH) free radical assay as described by Boly et al., 2016^17,20^. Briefly, we added 100 μL of freshly prepared DPPH reagent (0.1% in methanol) to 100 μL of the sample in a 96-well plate (*n* = 6) that we incubated at room temperature for 30 min in the dark. Next, we measured the reduction in DPPH color intensity at 540 nm using the microplate reader FluoStar Omega. We presented the data as mean ± standard deviation according to the following equation:

$$Percentage\,inhibition=\frac{Average\,absorbance\,of\,blank-Average\,absorbance\,of\,the\,test}{Average\,absorbance\,of\,blank}\times 100.$$

#### Assessment of the oxygen radical absorbance capacity (ORAC assay)

We prepared a 1 mM Trolox stock solution and performed nine serial dilutions to obtain 400, 300, 200, 150, 100, 75, 50, 25, and 12.5 μM solutions. We prepared a 400 µg/mL sample solution in MeOH. We performed the assay as described by Liang et al., 2014^22^, with minor modifications. Briefly, we incubated 12.5 μL of the prepared sample(s) with 75 μL of fluorescein (10 nM) for 30 min at 37 °C. Next, we measured background fluorescence (485 nm excitation and 520 nm emission) for three cycles (cycle time: 90 s). Then, we immediately added 12.5 μL of freshly prepared 2,2′-azobis(2-amidinopropane) (240 mM) to each well and continuously measured fluorescence (485 nm excitation and 520 nm emission) for 2.5 h (100 cycles of 90 s each). We presented the data as mean (*n* = 3) ± standard deviation and calculated the antioxidant effect of the extract as μM Trolox equivalents by substitution in the linear regression equation: Y = 32,356.3X + 989,769.9 (R^2^ = 0.9957).

### Assessment of cytotoxic activity

#### Cell culture

We obtained colorectal cancer (Colo-205), human melanoma (A-375), cervical cancer (HeLa), hepatocellular carcinoma (Hep G-2), large cell lung cancer (H-460), and oral epithelial (OEC) cells from Nawah Scientific Inc. (Mokatam, Cairo, Egypt). We maintained them in RPMI medium supplemented with 100 mg/mL of streptomycin, 100 units/mL of penicillin, and 10% of heat-inactivated fetal bovine serum and incubated them in humidified 5% (v/v) CO_2_ atmosphere at 37 °C^23^.

#### Cytotoxicity assay

We assessed cell viability through a sulforhodamine B (SRB) assay. We added 100 μL of cell suspension (5 × 10^3^ cells) to 96-well plates and incubated them in a complete medium for 24 h. We then treated the cells with 100 μL of medium containing samples at different concentrations (10 and 100 µg/mL). After 72 h of exposure, we fixed the cells by replacing the medium with 150 μL of 10% trichloroacetic acid and incubated them at 4 °C for 1 h. Next, we removed the trichloroacetic acid solution and washed the cells five times with distilled water. We then added 70 μL of SRB solution (0.4% w/v) and incubated the mixture in a dark place at room temperature for 10 min. We washed the plates three times with 1% acetic acid and allowed them to air-dry overnight. Then, we added 150 μL of TRIS (10 mM) to dissolve the protein-bound SRB stain and measured the absorbance at 540 nm using a BMG LABTECH-FLUOstar Omega microplate reader (Ortenberg, Germany)^23^.

#### Annexin-based apoptosis assay

Colo-205 cells were treated with either doxorubicin (10 µM) as a positive control or the total methanolic extract of *H. ramosissimum* for 48 h. Next, we collected the cells (10^5^ cells) by trypsinization and washed them twice with ice-cold phosphate-buffered saline (PBS, pH 7.4). We then incubated the cells in the dark with 0.5 mL of Annexin V-FITC/propidium iodide (PI) solution for 30 min at room temperature according to the manufacturer’s protocol (Annexin V-PI staining apoptosis detection kit from Abcam Inc., Cambridge Science Park, Cambridge, UK). After staining, we injected the cells into an ACEA Novocyte flow cytometer (ACEA Biosciences Inc., San Diego, CA, USA) and detected the FITC and PI fluorescent signals using FL1 and FL2 signal detectors, respectively (λ_ex/em_ = 488/530 nm for FITC and λ_ex/em_ = 535/617 nm for PI). For each sample, we acquired 12,000 events and quantified the FITC- and PI-positive cells by quadrant analysis using ACEA NovoExpress software (ACEA Biosciences Inc., San Diego, CA, USA)^24^.

#### Cell cycle distribution analysis

After treating the cells (10^5^ cells) with the total methanolic extract of *H. ramosissimum* for 48 h or paclitaxel (1 µM) for 24 h, as a positive control, we collected them by trypsinization and washed them twice with ice-cold PBS (pH 7.4). We then resuspended the cells in 2 mL of 60% ice-cold ethanol and incubated them at 4 °C for 1 h for fixation. Next, we washed the fixed cells twice with PBS (pH 7.4) and resuspended them in 1 mL of PBS containing 50 µg/mL RNAase A and 10 µg/mL PI. After 20 min of incubation in the dark at 37 °C, we analyzed the cells’ DNA contents by flow cytometry using an FL2 (λ_ex/em_ 535/617 nm) signal detector (ACEA Novocyte flow cytometer, ACEA Biosciences Inc., San Diego, CA, USA). For each sample, 12,000 events were acquired. We determined cell cycle distribution using ACEA NovoExpress software (ACEA Biosciences Inc., San Diego, CA, USA)^25^.

### Statistical analysis

We carried out triplicate experiments and analyzed data using *Microsoft Excel*. We determined the IC_50_ using *GraphPad Prism 5* by converting the concentrations to their logarithmic value and using the “non-linear inhibitor regression equation (log (inhibitor) vs. normalized response–variable slope equation)” function^26^.

### Molecular docking

We performed docking studies using Molecular Operating Environment (MOE, 2014.0901)^27^. We retrieved the three-dimensional (3D) structures of serologically defined colon cancer antigen 10 (PDB ID: 2HQ6) determined by X-ray diffraction (resolution: 1.75 Å) from the RCSB Protein Data Bank (https://www.rcsb.org/) and used it as a target in our molecular docking experiments^28^. We prepared and optimized the structure of the receptor protein using MOE’s ligx function with default settings. We drew the structures of the identified compounds in Chemdarw 17.0.0.206 in MOL format and performed 3D protonation, partial energy correction, and energy minimization using Merck molecular force field (MMFF94x). We determined the receptor active sites using MOE’s site finder tool. We carried out the molecular docking analysis to predict the receptor–ligand interaction using flexible ligand-fixed receptor docking parameters with Triangle Matcher placement (scoring: London dG; retain: 30) and force field refinement (rescoring: London dG; retain: 10). We selected the most stable protein–ligand interactions based on their S-score minimum energy and root mean square deviation (RMSD). We recorded the docking score, RSMD, and 2D and 3D interactions^29,30^.

## Results and discussion

### Phytochemical screening

We carried out phytochemical screening on *H. ramosissimum* to identify the different chemical classes of the total methanolic extract active constituents using different reagents. The preliminary screening revealed the presence of alkaloids, terpenoids, saponins, tannins, flavonoids, coumarins, polyphenolics, and reducing sugars.

### Total phenolic and flavonoid contents

We estimated the total phenolic content in the methanolic extract of *H. ramosissimum* to be 179.74 ± 0.58 µg/mL, in gallic acid equivalents. The equation for the standard curve was Y = 0.0031x − 0.0564 (Fig. S1), with R^2^ = 0.9961. Moreover, we measured a total flavonoid content of 53.18 ± 0.60 µg/mL, in rutin equivalents. The equation for the standard curve was Y = 0.0032x + 0.0398 (Fig. S2), with R^2^ = 0.9981.

### Metabolomic analysis

#### GC–MS analysis of the lipoidal matter

We performed a GC–MS analysis of the plant’s lipoidal matter to address the lack of information regarding its phytoconstituents. The GC–MS chromatogram (Fig. S3) of the lipoidal matter of *H. ramosissimum* revealed the presence of 41 compounds, and we identified 30 (91.73%) of them by comparing their mass spectra data (retention time and peak area) with the Wiley spectral library. We left 11 (8.25%) compounds unidentified. The most abundant compound was 1, 2-benzenedicarboxylic acid, bis (2-methoxyethyl) ester (23.91%), followed by 6,10,14-trimethylpentadecan-2-one (18.74%), ethyl hexadecanoate (13.68%), and hexadecanoic acid methyl ester (11.83%) (Table 1 and Fig. S4).

**Table 1:** GC–MS data of the *n*-hexane fraction of *H. ramosissimum* (Lehm.) DC. aerial parts.

Peak no	R_t_ (min)	Relative (%)	Mol. formula	[M^+^] *m/z*	Identified Compounds*	References
1	15.14	0.65	C_17_H_36_	240	2,6,10-Trimethyltetradecane	^31^
2	16.72	0.79	C_13_H_18_O_2_	206	3,4-Dihydro-2H-1,5-(3"-t-butyl) benzodioxepine	^32^
3	18.23	0.35	C_18_H_34_O_2_	282	Trans-13-octadecenoic acid	^33^
4	18.61	0.70	C_8_H_6_N_4_O_5_	238	2,4-Imidazolidinedione, 1-[[(5-nitro-2-furanyl) methylene]amino]-	^34^
5	19.86	0.39	C_16_H_30_O_4_Si3	370	Benzoic acid, 2,4-bis[(trimethylsilyl)oxy]-, trimethylsilyl ester	^35^
6	20.10	0.40	C_17_H_36_O	256	2-Methylhexadecan-1-ol	^36^
7	21.27	0.62	C_20_H_38_O_2_	310	cis-13-Eicosenoic acid	^37^
8	22.61	0.45	C_19_H_36_O_2_	296	cis-10-Nonadecenoic acid	^38^
9	22.74	0.55	C_32_H_66_	450	Dotriacontane	^39^
10	23.25	0.21	C_18_H_16_O_7_	344	4H-1-Benzopyran-4-one, 2-(3,4-dimethoxyphenyl)-3,5-dihydroxy-7-methoxy	^40^
11	23.73	18.74	C_18_H_36_O	268	6,10,14-Trimethylpentadecan-2-one	^41^
12	24.27	0.86	C_26_H_42_O_4_	418	1,2-Benzenedicarboxylic acid, tetradecyl ester	^42^
13	24.65	0.42	C_21_H_36_	288	14-α-Pregnane	^43^
14	24.76	0.22	C_18_H_34_O	266	11-Octadecenal	^44^
15	25.23	0.54	C_19_H_22_O_6_	346	Isochiapin B	^45^
16	22.35	11.83	C_17_H_34_O_2_	270	Hexadecanoic acid methyl ester	^46^
17	26.15	23.91	C_14_H_18_O_6_	282	1,2-Benzenedicarboxylic acid, bis (2-methoxyethyl) ester	^47^
18	26.66	13.68	C_18_H_36_O_2_	284	Ethyl hexadecanoate	^48^
19	27.28	0.51	C_18_H_36_O_2_	284	Methyl 14-methylheptadecanoate	^49^
20	28.54	2.51	C_19_H_34_O_2_	294	Linolelaidic acid, methyl ester	^50^
21	28.65	1.69	C_22_H_38_O_2_	334	Methyl 8-[2-((2-[(2-ethylcyclopropyl) methyl] cyclopropy) methyl) cyclopropyl] octanoate	^51^
22	29.12	1.78	C_19_H_38_O_2_	298	Methyl octadecanoate	^52^
23	29.73	1.26	C_20_H_36_O_2_	308	Linoleic acid ethyl ester	^48^
24	29.84	1.02	C_19_H_34_O_2_	294	(3E,12Z)-1,3,12-Nonadecatriene-5,14-diol	^53^
25	30.15	0.27	C_19_H_38_O_4_	330	2,3-Dihydroxypropyl palmitate	^54^
26	30.32	1.74	C_20_H_40_O_2_	312	Ethyl octadecanoate	^55^
27	31.59	0.51			Unknown	–
28	32.05	0.45	C_18_H_30_O_60_	268	2,2,3,3,4,4 Hexadeutero octadecanal	^56^
29	33.07	1.47			Unknown	–
30	33.68	0.46	C_21_H_40_O_2_	324	4,8,12,16-Tetramethylheptadecan-4-olide	^57^
31	33.94	0.45			Unknown	–
32	35.28	0.45			Unknown	–
33	36.11	2.42	C_24_H_38_O_4_	390	1,2-Benzenedicarboxylic acid, dioctyl ester	^58^
34	36.80	0.32			Unknown	–
35	37.91	0.79			Unknown	–
36	38.65	0.48			Unknown	–
37	38.82	0.42			Unknown	–
38	39.01	1.66			Unknown	–
39	39.14	2.31	C_29_H_50_O_4_	462	α-Tocospiro A	^58^
40	39.23	1.49			Unknown	–
41	39.58	0.21			Unknown	–

* The identification of *n*-hexane constituents based on the comparison of the mass spectral data with those of Mass Spectral Library (2011) and Wiley Registry of Mass Spectral Data 8th edition and literature.

#### LC–ESI–MS/MS profiling

We identified the compounds of the *H. ramosissimum* methanolic extract by LC–MS/MS. We compared their retention time (*R*_*t*_), mass, and MS2 with standards-reported literature, and databases (TMIC and MassBank). In total, we tentatively identified 32 compounds, including alkaloids, flavonoids, coumarins, phenolic acids, and their derivatives. We identified 17 compounds in negative mode and 15 in positive mode. Tables 2 and 3 list all identified compounds ordered by relative retention times and their structures were supplied (Figs. S5, S6, S74, S8).

**Table 2:** Metabolites identified in the methanolic extract of *H*. *ramosissimum* (Lehm.) DC. using positive mode LC–ESI–MS/MS.

No	R_t_ (min)	[M + H]^+^	Fragments	Identified Compound	Chemical Class	Ref
1	0.42	149	79, 105, 131	Cinnamic acid	Phenolic acids	^59^
2	9.28	191	115, 119, 147, 148	7-Methoxy-4-methylcoumarin	Coumarins	^59^
3	10.36	291	273, 139, 123	(epi)-Catechin	flavan -3-ol	^60^
4	10.94	300	138, 139, 157	Lycopsamine	Alkaloids	^61–63^
5	11.22	314	138, 156, 269	Heliospathine	Alkaloids	^61,62^
6	13.20	286	153, 127, 109	Trachelanthamine	Alkaloids	^64^
7	15.70	185	126, 80	Methyl gallate	Phenolics	^65^
8	15.95	277	134, 175, 217, 241	Stearidonic acid	Fatty acids	^66^
9	18.09	289	153, 127, 109	Eriodictyol	Flavonoids	^67^
10	19.57	287	241, 261, 213, 153	Kaempferol	Flavonoids	^68^
11	19.85	305	287, 259	Taxifolin	Flavonoids	^68^
12	19.89	330	121, 139, 241	Europine	Alkaloids	^61,69,70^
13	22.72	398	107, 121, 139	Heliosupine	Alkaloids	^61,71,72^
14	25.80	303	285, 275, 257, 229	Quercetin	Flavonoids	^68^
15	27.15	156	81, 112, 139	Retronecine	Alkaloids	^61,63,73,74^

**Table 3:** Metabolites identified in the methanolic extract of *H*. *ramosissimum* (Lehm.) DC. using negative mode LC–ESI–MS/ MS.

No	R_t_ (min)	[M-H]^−^	Fragments	Identified Compound	Chemical Class	Ref
1	2.21	115	97, 71	Maleic acid	Dicarboxylic acids	^59,75^
2	2.59	117	73, 98, , 116	Succinic acid	Dicarboxylic acids	^76^
3	4.14	181	125, 137, 153	2-Ethoxy-4,5-dihydroxybenzaldehyde	Phenolics	^77^
4	4.18	241	105, 195, 225	6-Deoxycochinolide	Benzofuran	^78^
5	4.48	175	113, 115, 131, 157	2-Isopropylmalic acid	Organic acids	^59,79^
6	4.61	138	79, 107, 110	7-Hydroxy-1-methylenepyrrolizidine	Alkaloids	^80^
7	4.70	153	153, 135, 123, 109	Protocatechuic acid	Phenolic acids	^59,81^
8	5.55	137	93, 109, 119	4-Hydroxybenzoic acid	Phenolic acids	^59,81,82^
9	5.6	179	135, 143, 161, 107	Caffeic acid	Phenolic acids	^83^
10	9.39	177	105, 133, 146, 176	4-Methoxycinnamic acid	Phenolic acids	^59^
11	10.01	289	243, 245	Filifolinoic acid	Phenolic acids	^84,85^
12	10.77	161	105, 117, 133	Umbelliferone	Coumarins	^59,86^
13	16.92	515	515, 353, 179, 173	4,5-di-*O*-caffeoylquinic acid (isochlorogenic acid)	Phenolic acids	^87^
14	20.05	337	337, 191, 163	3-*O*-*p*-coumaryl quinic acid	Phenolic acids	^87,88^
15	24.9	447	313, 319, 327, 357	Luteolin 8-*C*-Glucoside (Orientin)	Flavonoids	^89^
16	26.73	367	367, 191	3-*O*-feruloyl quinic acid	Phenolic acids	^90^
17	27.89	233	109, 121, 145, 175	Tournefolin C	Flavonoids	^77^

#### Free radical scavenging activity

The DPPH spectrophotometric assay is one of the most reliable and widely used methods to estimate the free radical scavenging effect of different plant extracts. The percent of inhibition values in the initial screening step of the two concentrations, namely, 1000 and 100 µg/mL, were 80.26 ± 1.50 (˃ 50) and 6.50 ± 0.73 (˂ 50), respectively. We serially diluted the extract that exceeded 50% inhibition (1000 µg/mL) to provide five concentrations that we tested to determine that the IC_50_ was 414.30 µg/mL (Fig. S9). For reference, Trolox has an IC_50_ of 24.42 ± 0.87 µM. The *H. ramosissimum* methanolic extract showed a potent radical-scavenging effect, which may be attributed to its total phenolic and flavonoid contents.

#### Assessment of ORAC assay

**Figure 2:**
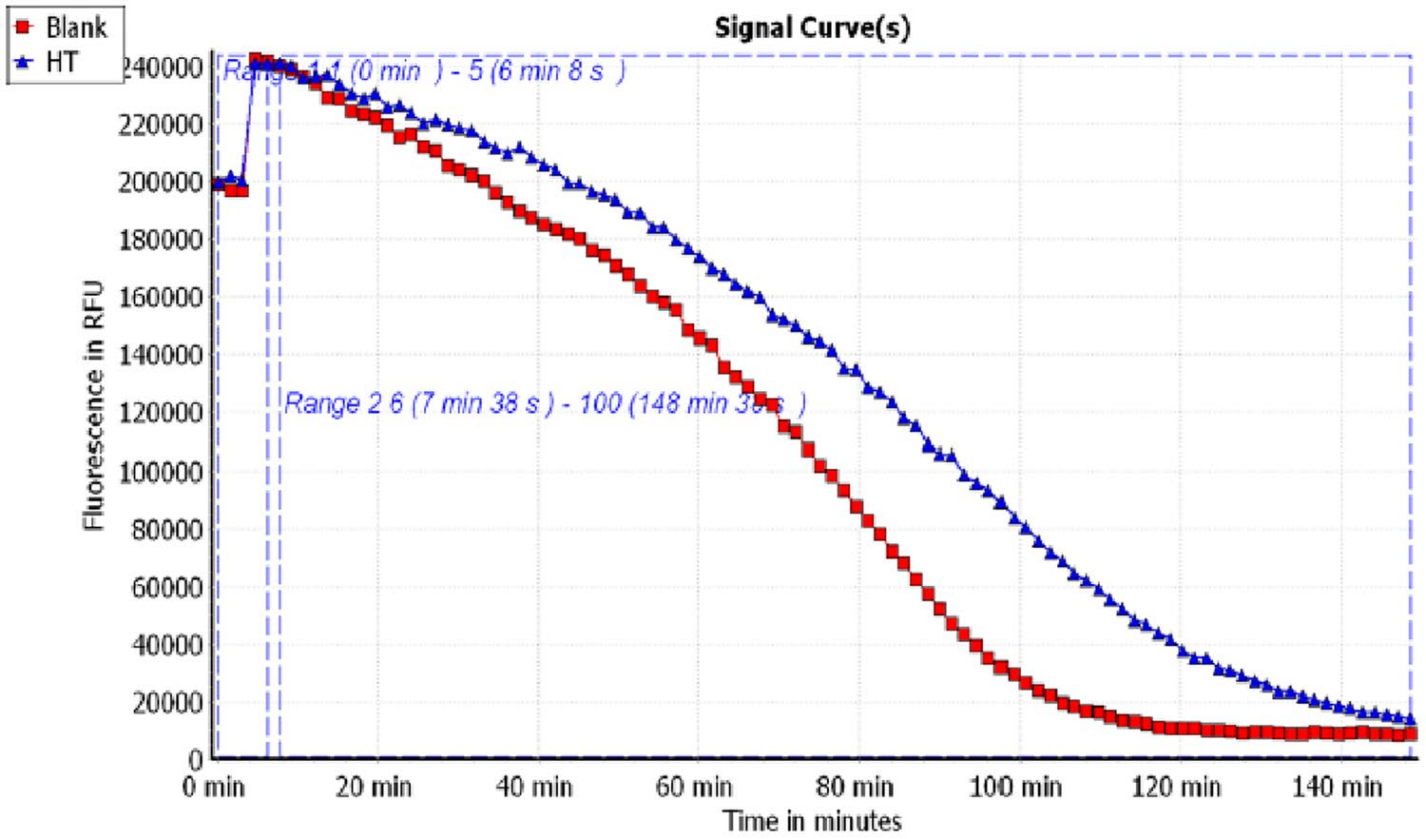
Signal curves of total methanolic extract of *H. ramosissimum* (Lehm.) DC. aerial parts (HT, blue color) and blank indicating the decay of fluoresceine upon applying the extract.

We evaluated the antioxidant activity of the *H. ramosissimum* methanolic extract using the ORAC assay (Fig. S10). The antioxidant activity of the extract (Fig. 2) was 170.03 ± 44.45 µM TE/equivalent, which is higher than that of Trolox.

#### Cytotoxicity assay

**Figure 3:**
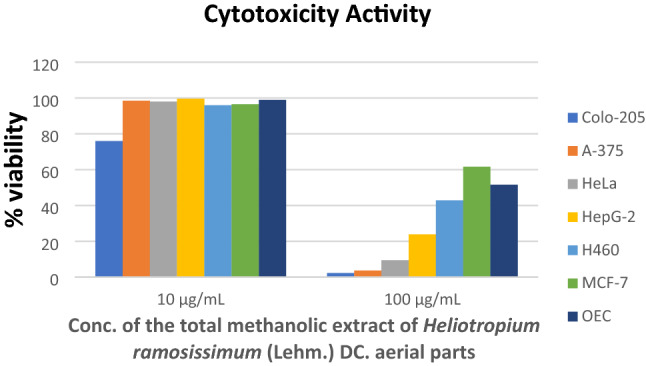
SRB cytotoxicity quick screening results of the total methanolic extract of *H. ramosissimum* (Lehm.) DC. aerial parts on different cell lines.

We screened the cytotoxicity of the *H. ramosissimum* methanolic extract at 10 and 100 µg/mL on various cell lines (Table 4 and Fig. 3). We performed an SRB quick screening assay on six cancer cell lines, namely, colorectal cancer (Colo-205), human melanoma (A-375), cervical cancer (HeLa), hepatocellular carcinoma (HepG-2), large cell lung cancer (H-460) cells, and a normal cell line, that is, oral epithelial cells (OEC). The *H. ramosissimum* methanolic extract exhibited potent activity on all tested cell lines. The most sensitive cell line was Colo- 205, followed by A-375, HeLa, and H-460, and the least sensitive was the MCF-7 cell line. Because the *H. ramosissimum* methanolic extract showed the highest activity against colorectal carcinoma (Colo-205) cells—in excellent agreement with the in silico study—we further investigated its IC_50_ and mechanism of action through cell cycle and apoptosis flow cytometry assays on this cell line.

**Table 4:** Cytotoxicity SRB quick screening results of the total methanolic extract of *H. ramosissimum* (Lehm.) DC. aerial parts.

Conc. of the tested sample	Cell viability %						
	Cancer cell lines						Normal cell line
	Colo-205	A-375	HeLa	HepG-2	H-460	MCF-7	OEC
10 µg/mL	75.95 ± 0.81	98.51 ± 0.44	98.03 ± 1.44	99.60 ± 0.19	95.93 ± 1.34	96.51 ± 0.73	98.94 ± 0.77
100 µg/mL	2.24 ± 0.37	3.65 ± 0.93	9.46 ± 0.37	23.82 ± 1.50	42.85 ± 0.26	61.66 ± 1.32	51.62 ± 0.48

± SD (n = 3).

**Figure 4:**
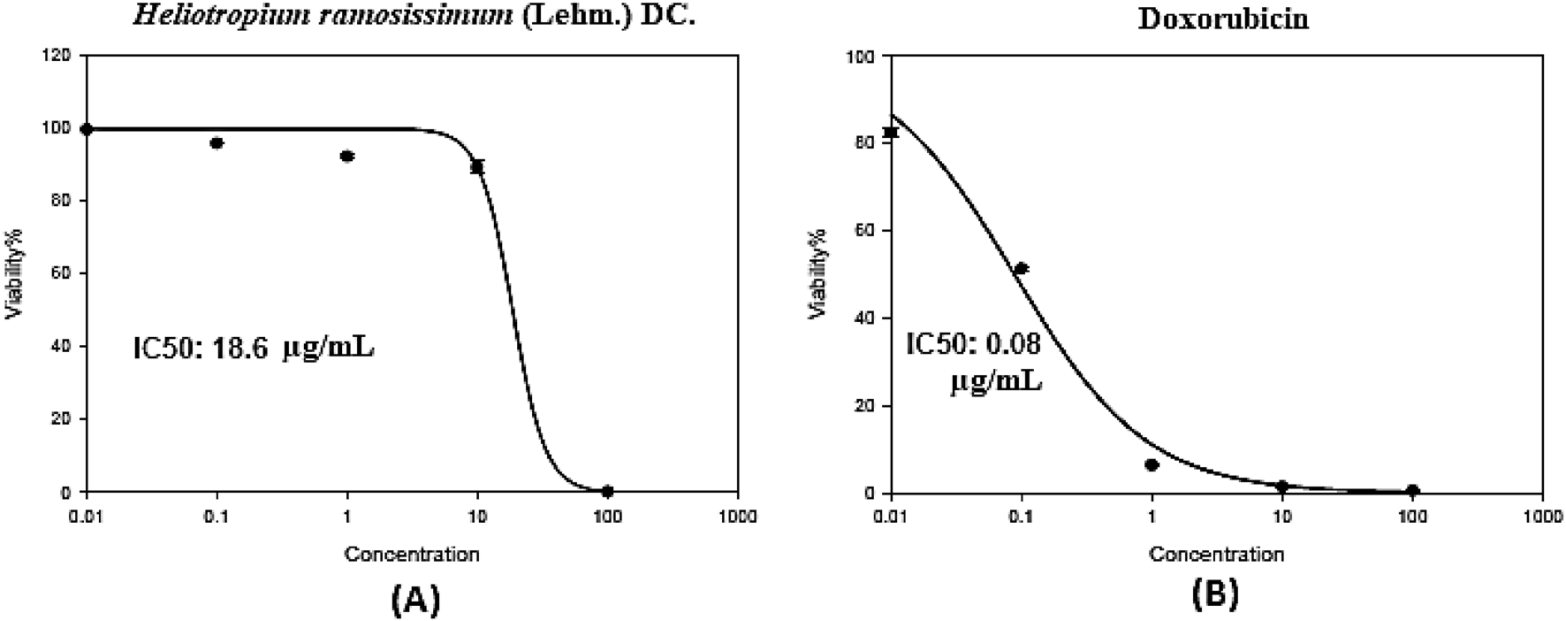
*In-vitro* SRB cytotoxicity assay of (**A**) the total methanolic extract of *H. ramosissimum* (Lehm.) DC. aerial parts; (**B**) Doxorubicin; in increasing concentrations (0.01–100 µg/mL) against colorectal carcinoma (Colo-205) cell lines. Data points are expressed as mean ± SD (n = 3).

**Figure 5:**
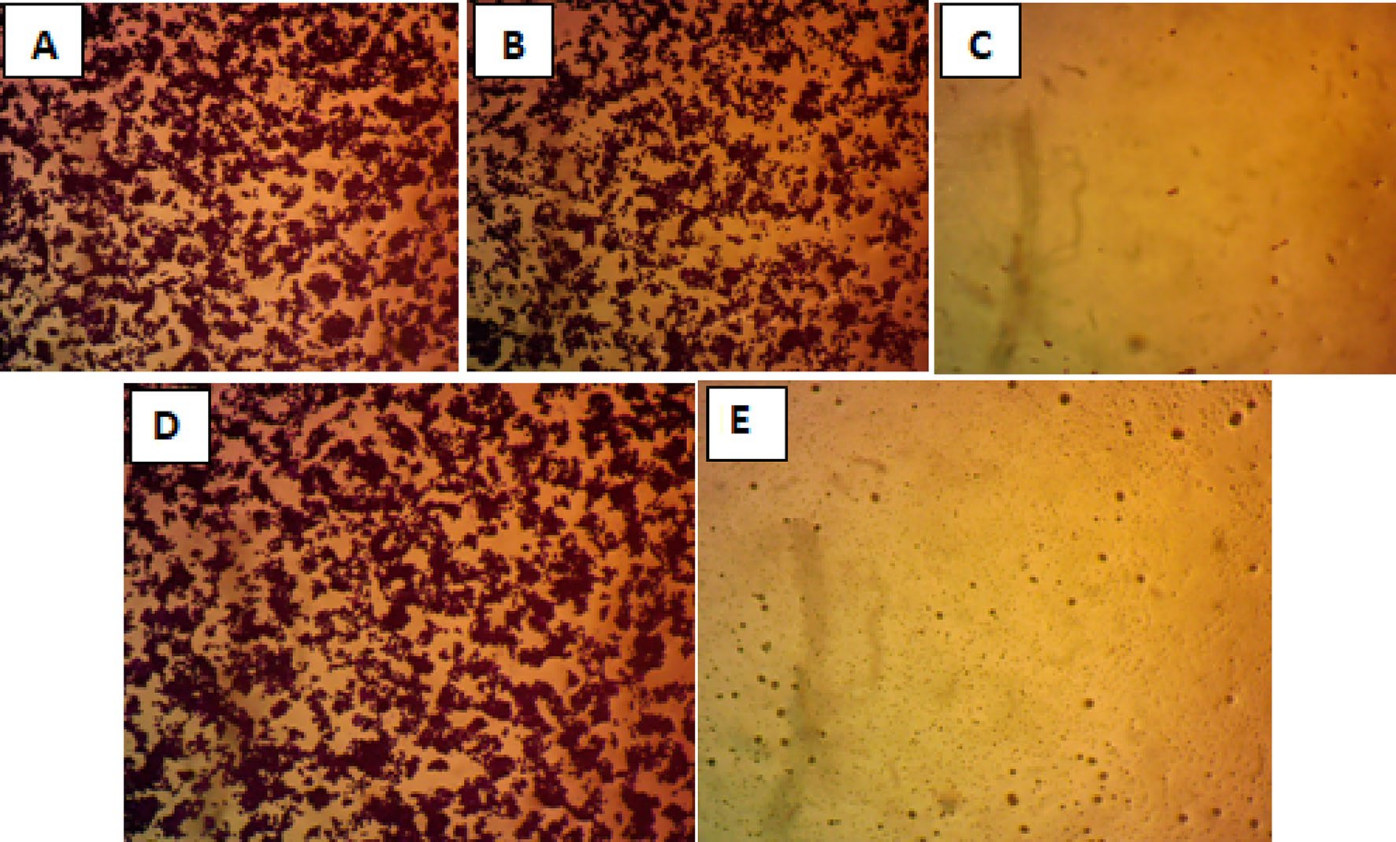
Optical microscope stained images of SRB cytotoxicity assay against Colo-205 cell line (**A**) Negative control, (**B**) Doxorobucin (0.01 µg/mL), (**C**) Doxorobucin (100 µg/mL), (**D**) the total methanolic extract of *H. ramosissimum* (Lehm.) DC. aerial parts (0.01 µg/mL), (**E**) the total methanolic extract of *H. ramosissimum* (Lehm.) DC. aerial parts (100 µg/mL), magnification power: X 100.

We assessed cell viability at five different concentrations (0.01, 0.1, 1, 10, and 100 µg/mL) and used doxorubicin as a standard cytotoxic drug. The SRB assay (Figs. 4, 5) revealed that the *H. ramosissimum* methanolic extract possesses a dose-dependent cytotoxic effect with an IC_50_ of 18.60 µg/mL (Fig. 4A) and that of doxorubicin is 0.08 µg/mL (Fig. 4B). The *H. ramosissimum* extract decreased cell viability from 75.95% ± 0.81% at 10 µg/mL to 2.24% ± 0.37% at 100 µg/mL (Table 4).

The optical microscope staining images (Fig. 5) show the results of the SRB cytotoxicity assay against Colo-205 cell line of both total Colo-205 cells for two *H. ramosissimum* methanolic extract concentrations (0.01 and 100 µg/mL), doxorubicin at the same concentrations, and negative control. The figure clearly shows that at 0.01 µg/mL, neither the extract nor doxorubicin caused significant morphological changes. Meanwhile, significant changes occurred at 100 µg/mL, confirming the dose-dependent character of the cytotoxicity of the extract. To investigate the safety of the *H. ramosissimum* methanolic extract on normal cells and the selectivity of its cytotoxicity on cancer cells, we performed the cytotoxic activity assay on OECs. The viability of OECs treated with 10 µg/mL methanolic extract was 98.94% ± 0.77%. Meanwhile, this concentration showed a potent cytotoxic effect on Colo-205 cells by reducing cell viability to 75.95% ± 0.81%.

**Figure 6:**
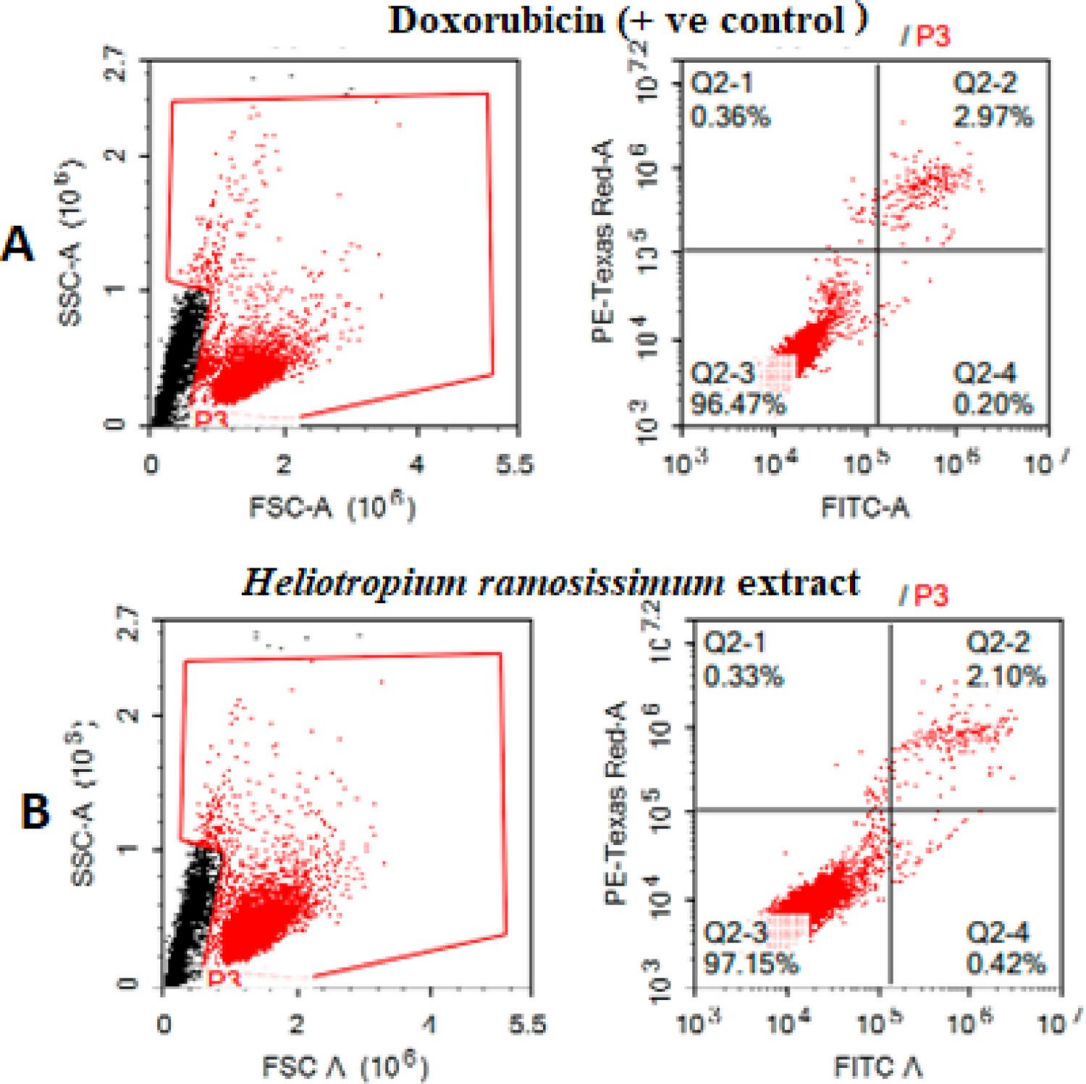
Apoptosis in Colo-205 cells estimated by Annexin V and PI staining followed by flow cytometry analysis. Quadrants represent dead (Q1), late apoptotic (Q2), live (Q3), and (Q4) early apoptotic cells. (**A**) Control, (**B**) the total methanolic extract of *H. ramosissimum* (Lehm.) DC. aerial parts.

To further investigate the mechanism responsible for the cytotoxic activity of the *H. ramosissimum* methanolic extract, we carried out an apoptosis assay on Colo-205 cells. We used Annexin V-FITC/PI double staining and evaluated the apoptotic rates using flow cytometry. As illustrated in Fig. 6, the apoptotic rate was 56.14% in the positive control group using doxorubicin, whereas treating the cells with 18 µg/mL of methanolic extract (which is equal to its IC_50_) significantly increased the apoptotic rate to 71.76%. These results suggest that the extract reduced Colo205 cell viability by inducing apoptosis.

**Figure 7:**
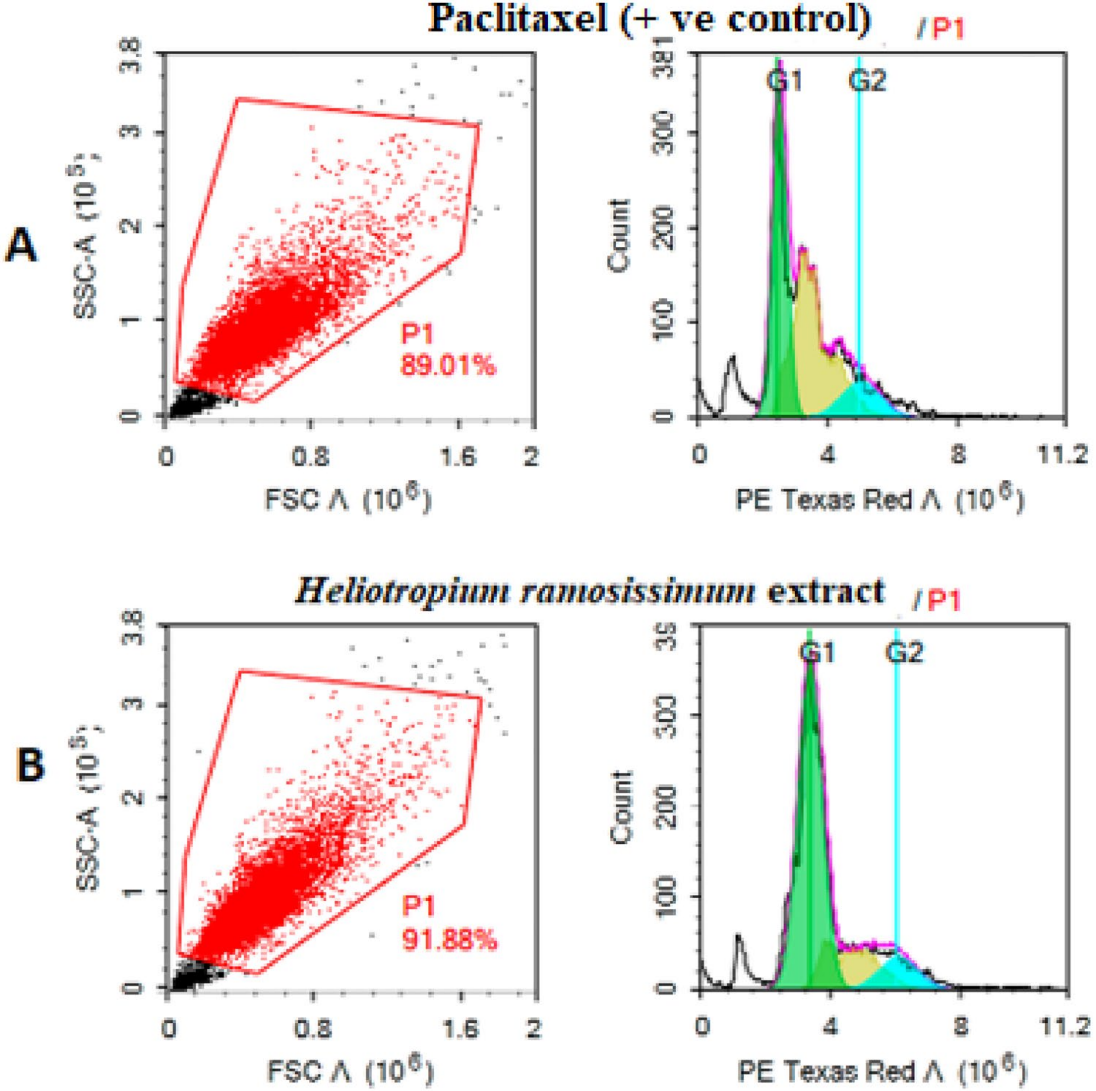
Schematic representation of cell cycle arrest at Sub G1 and G2 phase (**A**) Control, (**B**) the total methanolic extract of *H. ramosissimum* (Lehm.) DC. aerial parts.

To examine whether the cytotoxicity of the *H. ramosissimum* methanolic extract on Colo-205 was associated with cell cycle arrest, we conducted a PI-metric cell cycle analysis using flow cytometry (Fig. 7). The cell cycle histograms revealed that cells treated with the extract (18 µg/mL) had a markedly higher sub-G1 population (63.01%) than those treated with the positive control, paclitaxel (37.33%). This suggests that the extract induced apoptosis and/or necrosis in Colo-205 cells.

We reviewed the literature on the cytotoxic activity of different *Heliotropium* species and found that the *n*-hexane fraction of the ethanolic extract of *H. subulatum* aerial parts displayed cytotoxic activity at 3 mg/mL^91^. An in vitro assay against MRC5 human cells revealed that the methanolic extract of *H. zeylanicum* aerial parts exerted significant cytotoxicity with an IC_50_ of 13 µg/mL^92^. Furthermore, the methanolic extract of the dried roots of *H. indicum* showed different mortality rates at different concentrations, with a lethal concentration (LC_50_) of 47.86 μg/mL and LC_90_ of 75.85 μg/mL in a brine shrimp lethality bioassay^93^. A 3-(4,5-dimethylthiazol-2-yl)-2,5-diphenyltetrazolium bromide (MTT) assay revealed that the whole plant ethanolic extract of *H. indicum* had significant antiproliferative activity against SKBR-3 human breast adenocarcinoma cells. The IC_50_ of the extract was 34 ± 9.09 μg/mL, and that of the standard drug paclitaxel was 22.20 ± 2.30 μg/mL^94^. These findings suggest that the *H. ramosissimum* methanolic extract exerts a potent cytotoxic effect, especially against colorectal carcinoma, by inducing apoptosis. These results suggest that this extract is a promising candidate for the treatment of this type of cancer.

### Molecular docking

In silico molecular docking, techniques have enhanced drug discovery and development by allowing the structure-based exploration of ligand-receptor interactions. The cytotoxicity assays revealed that the cytotoxicity of the methanolic extract was more potent against colon cancer than against the other cell lines. We investigated the possible interactions between the 32 phytochemical compounds identified by LC–ESI–MS/MS and the colon cancer antigen 10 binding sites and compared them to the standard anticancer drug doxorubicin. Table 5 shows the binding energies of the compounds. The computed binding score energy values range from − 6.4244 kcal/mol (hydroxybenzoic acid) to − 14.0088 kcal/mol (doxorubicin).

**Table 5:** Docking scores of identified compounds against colon cancer antigen 10 (PDB ID: 2HQ6).

No	Compound	Binding score (kcal/mol)	RSMD_refine (Å)	E_conf (kcal/mol)
1	Cinnamic acid	− 6.7353	1.07	− 75.77
2	Methyl gallate	− 8.4939	1.36	− 99.08
3	Taxifolin	− 11.2287	1.32	− 17.49
4	7-Methoxy-4-methylcoumarin	− 6.5481	1.12	34.50
5	Quercetin	− 10.6524	0.92	35.93
6	epicatechin	− 10.6177	1.26	23.60
7	Catechin	− 10.3107	1.28	23.49
8	Lycopsamine	− 8.8984	1.89	9.61
9	Heliospathine	− 7.9719	0.88	− 7.98
10	Trachelanthamine	− 8.5378	1.44	18.25
11	Protocatechuic acid	− 8.0570	0.98	− 71.46
12	Stearidonic acid	− 7.8434	1.81	− 53.25
13	Eriodictyol	− 10.5128	1.31	9.94
14	Kaempferol	− 11.8367	0.66	− 52.05
15	Europine	− 9.2788	1.29	39.07
16	Heliosupine	− 8.5358	1.06	117.43
17	Retronecine	− 7.7943	1.82	− 60.09
18	Maleic acid	− 7.1007	0.66	− 177.99
19	Caffeic acid	− 9.5269	1.34	− 96.22
20	2-Ethoxy-4,5-dihydroxybenzaldehyde	− 9.6644	0.65	16.05
21	6-Deoxycochinolide	− 8.2384	0.89	− 1.10
22	2-Isopropylmalic acid	− 9.5676	0.84	− 36.78
23	7-Hydroxy-1-methylenepyrrolizidine	− 7.5278	0.90	4.06
24	4-Hydroxybenzoic acid	− 6.4245	1.48	− 68.26
25	Succinic acid	− 6.8769	0.83	− 176.13
26	4-Methoxycinnamic acid	− 7.3200	1.52	− 72.06
27	Filifolinoic acid	− 8.7365	0.84	3.57
28	Umbelliferone	− 9.2712	1.42	12.29
29	4,5-di-*O*-caffeoylquinic acid (Isochlorogenic Acid)	− 13.2001	1.46	− 4.03
30	3-*O*-*p*-coumarylquinic acid	− 9.5033	1.09	10.87
31	3-*O*-Feruloylquinic acid	− 10.1228	0.85	23.34
32	Orientin	− 13.5655	1.16	53.38
33	Tournefolin C	− 9.0987	0.97	9.53
34	Doxorubicin	− 14.0088	1.71	− 79.03

**Figure 8:**
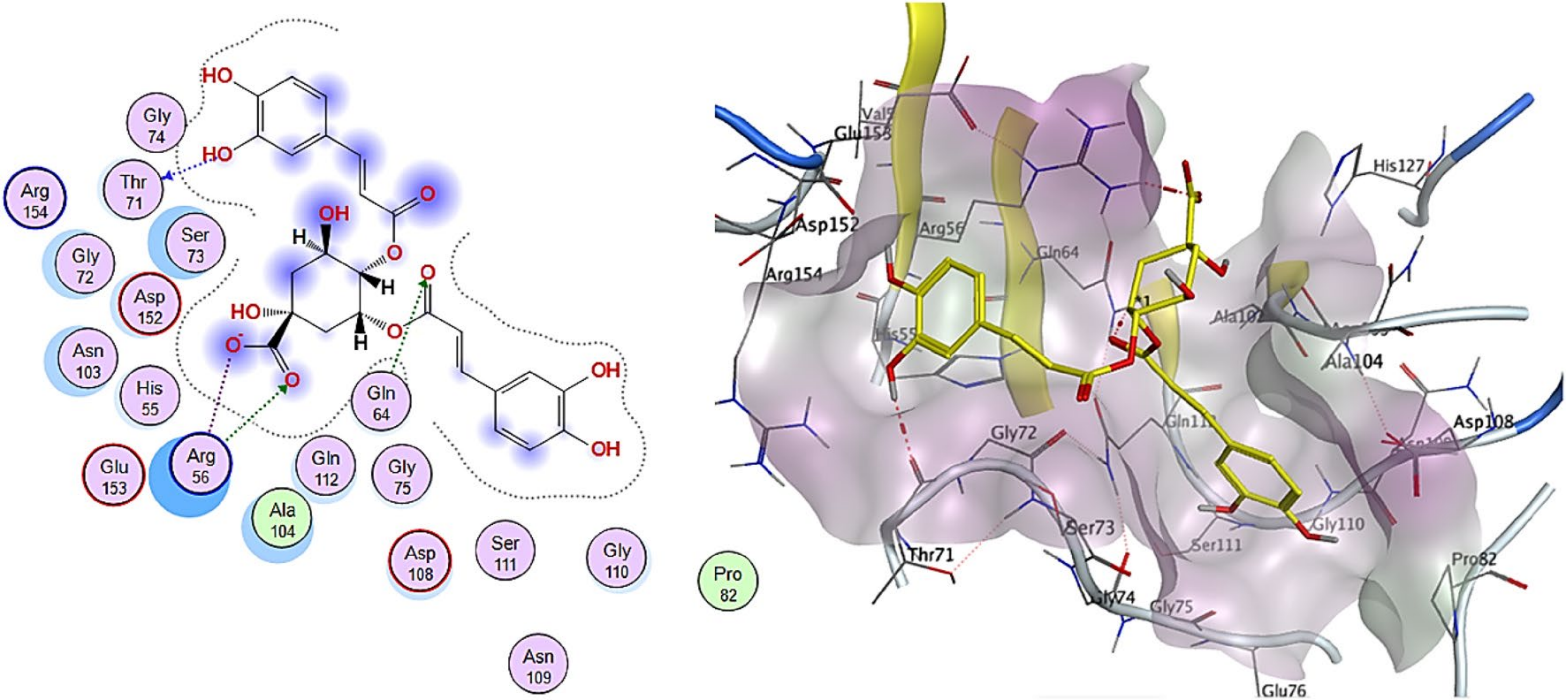
2D and 3D interactions complex of Isochlorogenic acid against colon cancer antigen 10 (generated by using Molecular Operating Environment, MOE, 2014.0901^99^).

**Figure 9:**
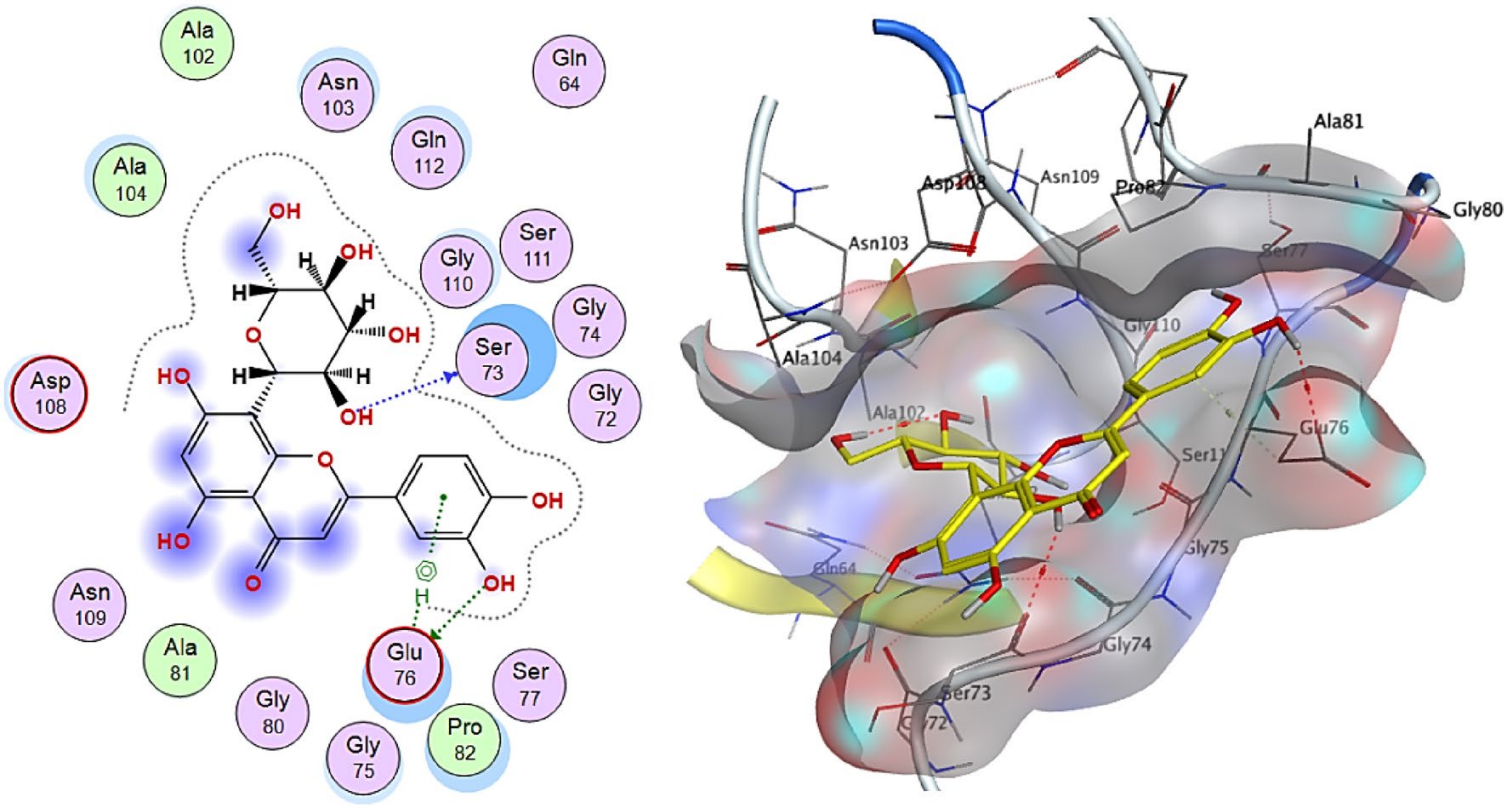
2D and 3D interactions complex of Orientin against colon cancer antigen 10 (generated by using Molecular Operating Environment, MOE, 2014.0901^99^).

Isochlorogenic acid, orientin, kaempferol, and taxifolin are the top-scoring compounds with pose scoring values of − 13.200103, − 13.5655, − 11.836662, and − 11.228658 kcal/mol, respectively. Isochlorogenic acid and orientin (flavonol glycoside) showed the best interactions with the receptor, with the lowest binding energy values of − 13.2001 kcal/mol (RMSD = 1.46 Å) and − 13.5655 kcal/mol (RMSD = 1.16 Å), respectively. These values are close to that of doxorubicin (− 14.0088 kcal/mol, RMSD = 1.71 Å), indicating that they could inhibit the receptor. Isochlorogenic acid interaction with the receptor involved hydrogen bonds with Thr71 (H-donor), Gln64 (H-acceptor), and Arg56 (H-acceptor) in addition to ionic interactions with Arg 56AA (Fig. 8). Orientin formed interactions with Ser73AA (H-donor) and π-H interaction with Glu 76 (Fig. 9).

**Figure 10:**
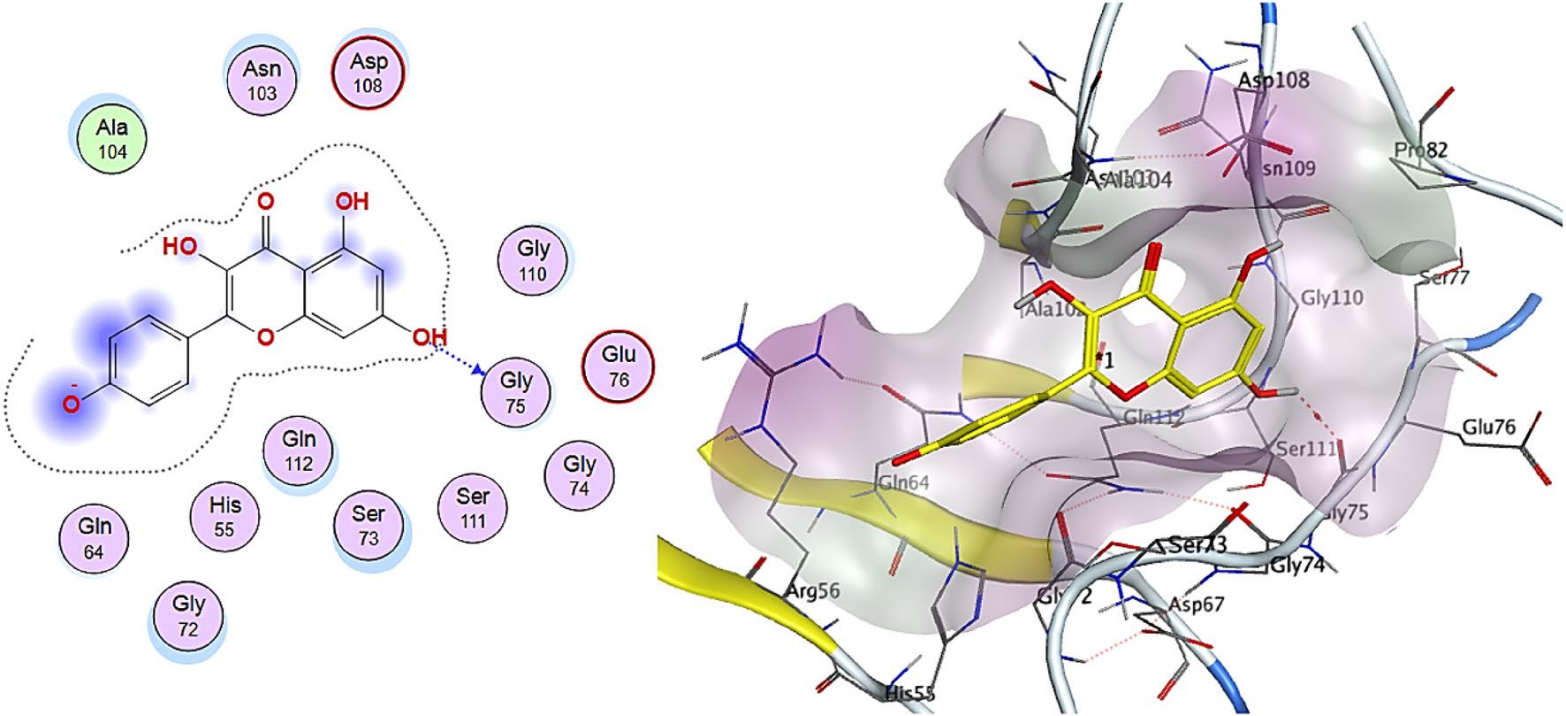
2D and 3D interactions complex of Kaempferol against colon cancer antigen 10 (generated by using Molecular Operating Environment, MOE, 2014.0901^99^).

**Figure 11:**
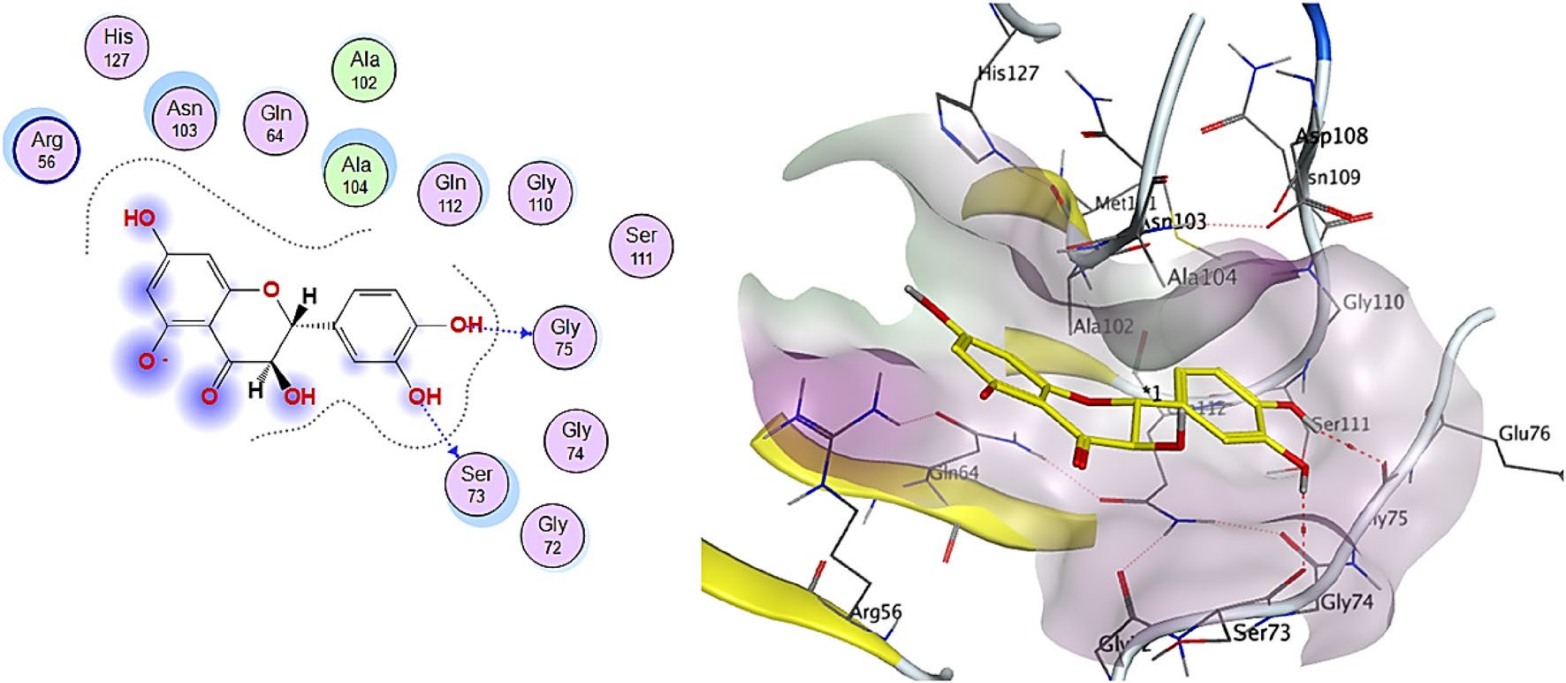
2D and 3D interactions complex of Taxifolin against colon cancer antigen 10 (generated by using Molecular Operating Environment, MOE, 2014.0901^99^).

Kaempferol (Fig. 10) formed hydrogen bonds with Gly75AA (H-donor), and taxifolin (Fig. 11) interacted with Gly75AA and Ser73AA (H-donor). The cytotoxicity of the tested extract may be attributed to the phenolic compounds and flavonoids with the best binding affinity in the molecular docking study.

The previous experimental studies are matched with our molecular docking results. Chlorogenic acid was reported to exhibit potential effects on cytotoxicity and inhibited human colon cancer cell proliferation through cell-cycle arrest and apoptosis^95^. In addition, orientin exhibited remarkable cytotoxicity and antiproliferative activity against HT-29 colon cancer cells, induced G0/G1 cell cycle arrest, regulated cyclin and cyclin-dependent protein kinases, and mediated apoptosis in human colorectal cancer HT-29 cells^96^. Further, taxifolin showed human colorectal cancer cell growth arrest in the G2 phase of the cell cycle and apoptosis in a concentration-dependent approach^97^. Besides, kaempferol's cytotoxic effects and induction of apoptosis in different human colorectal cancer cell lines have been reported^98^.

## Conclusion

In conclusion, the phytochemical screening of the total methanolic extract of the aerial parts of *H. ramosissimum* (Lehm.) DC. revealed the presence of alkaloids, terpenoids, saponins, tannins, flavonoids, coumarins, polyphenolics, and reducing sugars. It is worthy to mention that all the identified compounds in the *n*-hexane fraction are the first to be reported in the genus *Heliotropium*, in addition, thirty-two compounds of different chemical classes were tentatively identified by LC–ESI–MS/MS analysis. Besides, our findings revealed that the total methanolic extract of *H. ramosissimum* aerial parts exhibits a potent in vitro antitumor effect against several cell lines especially colorectal carcinoma (Colo-205) in a dose-dependent manner and with selectivity in comparison to normal cell lines (OEC). The methanolic extract reduces the viability of invasive Colo-205 cell lines by inducing apoptosis and/or necrotic cell death. Furthermore, a docking study for the identified compounds from the LC–ESI–MS/MS analysis to find out the candidates responsible for the cytotoxic activity where isochlorogenic acid and orientin showed the highest binding scores with colon cancer antigen 10. Our findings can aid in the creation of a new alternative candidate for the selective treatment of early stages of colorectal cancer with safety on normal cells. Future studies are needed to isolate the active constituent/constituents responsible for this potent cytotoxic activity.

## Supplementary Information

Supplementary Figures.

## Data Availability

All data generated or analyzed during this study are included in this published article [and its supplementary information files].

## Abbreviations

A-375: Human melanoma cell line
AlCl_3_: Aluminum chloride
Colo-205: Colorectal cancer cell line
DPPH: 2,2-Diphenyl-1-picrylhydrazyl
GAE: Gallic acid equivalents
GC-MS: Gas chromatography-mass spectrometry
H-460: Large cell lung cancer cell line
HeLa: Cervical cancer cell line
HepG-2: Hepatocellular carcinoma cell line
LC–ESI–MS/MS: Liquid chromatography–electrospray ionization–mass spectrometry
OEC: Oral epithelial cell line
ORAC: Oxygen radical absorbance capacity
RE: Rutin equivalents
SRB: Sulforhodamine B
TE: Trolox equivalent

## References

[1] Riedl H (1967). Boraginaceae.

[2] Dash G, Abdullah M (2013). A review on *Heliotropium indicum* L. (Boraginaceae). Int. J. Pharm. Sci. Res..

[3] Thulin M (1993). Flora of Somalia, Volume 1-Pteridophyta; Gymnospamae; angiospamae (Annonacae-Fabaceae).

[4] Schmelzer GH, Gurib-Fakim A (2008). Plant Resource of Tropical Africa 11 (1) Medicinal Plants 1.

[5] Ayensu ES (1978). Medicinal Plants of West Africa.

[6] Nagaraju N, Rao K (1990). A survey of plant crude drugs of Rayalaseema, Andhra Pradesh, India. J. Ethnopharmacol..

[7] Wiart C (2006). Medicinal Plants of the Asia-Pacific: Drugs for the Future?.

[8] Roeder E, Wiedenfeld H (2009). Pyrrolizidine alkaloids in medicinal plants of Mongolia, Nepal and Tibet. Pharmazie.

[9] Neuwinger HD (2000). African Traditional Medicine: A Dictionary of Plant Use and Applications. With Supplement: Search System for Diseases.

[10] Qureshi R, Bhatti GR (2008). Ethnobotany of plants used by the Thari people of Nara Desert, Pakistan. Fitoterapia.

[11] Fayed MA (2021). Heliotropium; a genus rich in pyrrolizidine alkaloids: A systematic review following its Phytochemistry and Pharmacology. Phytomed. Plus.

[12] Scienc, R. B. F. K. *Heliotropium ramosissimum*. http://plantsoftheworldonline.org/taxon/urn:lsid:ipni.org:names:117174-1 (2020).

[13] Khan MA, Khan AS (1980). Isolation of heliotrine N-oxide from *Heliotropium ramosissimum*. Planta Med..

[14] Shahat AA, Ibrahim AY, Elsaid MS (2014). Polyphenolic content and antioxidant activity of some wild Saudi Arabian Asteraceae plants. Asian Pac. J. Trop. Med..

[15] Abdou EM, Fayed MAA, Helal D, Ahmed KA (2019). Assessment of the hepatoprotective effect of developed lipid-polymer hybrid nanoparticles (LPHNPs) encapsulating naturally extracted β-Sitosterol against CCl_4_ induced hepatotoxicity in rats. Sci. Rep..

[16] Bakr RO (2021). *In-vivo* wound healing activity of a novel composite sponge loaded with mucilage and lipoidal matter of Hibiscus species. Biomed. Pharmacother..

[17] El-Shanawany MA, Sayed HM, Ibrahim SRM, Fayed MAA (2014). Chemical constituents, anti-inflammatory, and antioxidant activities of *Anisotes trisulcus*. Bull. Fac. Pharm. Cairo Univ..

[18] Radhia A, Hanen N, Abdelkarim BA, Mohamed N (2018). Phytochemical screening, antioxidant and antimicrobial activities of *Erodium glaucophyllum* (L.) L'Hérit. J. Biomed. Sci..

[19] Thilagavathi T, Arvindganth R, Vidhya D, Dhivya R (2015). Preliminary phytochemical screening of different solvent mediated medicinal plant extracts evaluated. Int. Res. J. Pharm.

[20] Boly R, Lamkami T, Lompo M, Dubois J, Guissou I (2016). DPPH free radical scavenging activity of two extracts from *Agelanthus dodoneifolius* (Loranthaceae) leaves. Int. J. Toxicol. Pharmacol. Res..

[21] Mostafa E, Fayed MAA, Radwan RA, Bakr RO (2019). *Centaurea pumilio* L. extract and nanoparticles: A candidate for healthy skin. Colloids Surf. B..

[22] Liang Z, Cheng L, Zhong G-Y, Liu RH (2014). Antioxidant and antiproliferative activities of twenty-four *Vitis vinifera* grapes. PLoS ONE.

[23] Basiouni S (2020). Characterization of sunflower oil extracts from the Lichen *Usnea barbata*. Metabolites.

[24] Venghateri JB, Gupta TK, Verma PJ, Kunwar A, Panda D (2013). Ansamitocin P3 depolymerizes microtubules and induces apoptosis by binding to tubulin at the vinblastine site. PLoS ONE.

[25] Ahmed RM, Fayed MAA, El-Behairy MF, Abdallah IA (2020). Identification, isolation, structural characterization, *in silico* toxicity prediction and *in vitro* cytotoxicity assay of simeprevir acidic and oxidative degradation products. RSC Adv..

[26] Chen Z, Bertin R, Froldi G (2013). EC50 estimation of antioxidant activity in DPPH assay using several statistical programs. Food Chem..

[27] Vilar S, Cozza G, Moro S (2008). Medicinal chemistry and the molecular operating environment (MOE): Application of QSAR and molecular docking to drug discovery. Curr. Top. Med. Chem..

[28] Diab M (2019). Inner metal complexes of tetradentate Schiff base: Synthesis, characterization, biological activity and molecular docking studies. Appl. Organomet. Chem..

[29] Arif R (2021). Molecular docking and simulation studies of antidiabetic agents devised from hypoglycemic polypeptide-P of *Momordica charantia*. BioMed Res. Int..

[30] Mahnashi MH (2021). Phytochemical profiling of bioactive compounds, anti-inflammatory and analgesic potentials of *Habenaria digitata* Lindl.: Molecular docking based synergistic effect of the identified compounds. J. Ethnopharmacol..

[31] Achakzai JK (2019). *In vitro* antileishmanial activity and GC-MS analysis of whole plant hexane fraction of *Achillea wilhelmsii* (WHFAW). J. Chem..

[32] Nagella P, Ahmad A, Kim SJ, Chung IM (2012). Chemical composition, antioxidant activity and larvicidal effects of essential oil from leaves of *Apium graveolens*. Immunopharmacol. Immunotoxicol..

[33] Krishnamoorthy K, Subramaniam P (2014). Phytochemical profiling of leaf, stem, and tuber parts of *Solena amplexicaulis* (Lam.) Gandhi using GC-MS. Int. Sch. Res. Not..

[34] Ghalloo BA (2022). Phytochemical profiling, *in vitro* biological activities, and *in silico* molecular docking studies of *Dracaena reflexa*. Molecules.

[35] Wang GJ, Tian L, Fan YM, Qi ML (2013). Headspace single-drop microextraction gas chromatography mass spectrometry for the analysis of volatile compounds from Herba Asari. J. Anal. Methods Chem..

[36] Generalić Mekinić I (2021). Seasonal changes in essential oil constituents of *Cystoseira compressa*: First report. Molecules.

[37] Avato P, Rosito I, Papadia P, Fanizzi FP (2006). Characterization of seed oil components from *Nephelium Lappaceum* L.. Nat. Prod. Commun..

[38] Jiang J, Jia X (2015). Profiling of fatty acids composition in suet oil based on GC-EI-qMS and chemometrics analysis. Int. J. Mol. Sci..

[39] Gurning, K., Iksen, I., Simanjuntak, H. A. & Purba, H. Identification of the chemical compound of essential oil from Ketumbar (*Coriandrum sativum* L) leaves with GC-MS. *Pharmacogn. J.* **12**, 1019–1023 (2020).

[40] Saravanan R, Raja K, Shanthi D (2022). GC–MS analysis, molecular docking and pharmacokinetic properties of phytocompounds from *Solanum torvum* unripe fruits and its effect on breast cancer target protein. Appl. Biochem. Biotechnol..

[41] Abu-Lafi S, Rayan B, Kadan S, Abu-Lafi M, Rayan A (2019). Anticancer activity and phytochemical composition of wild *Gundelia tournefortii*. Oncol. Lett..

[42] Ghalloo BA (2022). Phytochemical profiling, *in vitro* biological activities, and *in silico* molecular docking studies of *Dracaena reflexa*. Molecules (Basel, Switzerland).

[43] Kartal M, Kaya S, Kurucu S (2002). GC-MS analysis of propolis samples from two different regions of Turkey. Zeitschrift fur Naturforschung. C. J. Biosci..

[44] Zellagui A, Gherraf N, Ladjel S, Hameurlaine S (2012). Chemical composition and antibacterial activity of the essential oils from *Launaea resedifolia* L. Org. Med. Chem. Lett..

[45] Piva RC, Verdan MH, Branquinho LS, Kassuya CAL, Cardoso CAL (2021). Anti-inflammatory activity and chemical composition of aqueous extract and essential oil from leaves of *Ocimum selloi* Benth. J. Ethnopharmacol..

[46] Zayed MZ, Ahmad FB, Ho W-S, Pang S-L (2014). Gc-Ms analysis of phytochemical constituents in leaf extracts of *Neolamarckia Cadamba* (Rubiaceae) from Malaysia. Int. J. Pharm. Pharm. Sci..

[47] Efiom OO (2010). Isolation and characterization of bis (2-Methoxyethyl) phthalate and hexashydro-1-3-dimethyl-4-phenyl-1h-azepine 4-carboxylic acid from the root of Cissampelos owariensis (*P. Beauv*). Niger. J. Basic Appl. Sci..

[48] Shah M (2021). GC-MS analysis and biomedical therapy of oil from n-hexane fraction of *Scutellaria edelbergii* Rech. f.: In vitro, in vivo, and in silico approach. Molecules (Basel, Switzerland).

[49] Alhassan AJ, Sule MS (2013). GC–MS characterization of degutted white grubs’ fatty acids composition. Chem. Search J..

[50] Shen C-Y, Zhang T-T, Zhang W-L, Jiang J-G (2016). Anti-inflammatory activities of essential oil isolated from the calyx of *Hibiscus sabdariffa* L. Food Funct..

[51] Odutayo OE, Omonigbehin EA, Olawole TD, Ogunlana OO, Afolabi IS (2020). Fermentation enhanced biotransformation of compounds in the kernel of *Chrysophyllum albidum*. Molecules (Basel, Switzerland).

[52] Abubakar MN, Majinda RRT (2016). GC–MS analysis and preliminary antimicrobial activity of *Albizia adianthifolia* (Schumach) and *Pterocarpus angolensis* (DC). Medicines (Basel, Switzerland).

[53] Venceslau ADFA (2021). Analysis of the chemical constituents of *Thompson atemoya* seed oil. Rev. Bras. Frutic..

[54] Jahan I (2020). GC-MS phytochemical profiling, pharmacological properties, and *in silico* studies of *Chukrasia velutina* leaves: A novel source for bioactive agents. Molecules (Basel, Switzerland).

[55] Politi L, Mari F, Furlanetto S, Del Bravo E, Bertol E (2011). Determination of fatty acid ethyl esters in hair by GC-MS and application in a population of cocaine users. J. Pharm. Biomed. Anal..

[56] Lalitha G, Nazeema TH, Anitha P (2019). GC–MS analysis of bioactive components on the leaves extract of *Elaeagnus conferta* Roxb.. Int. Res. J. Pharm..

[57] Rontani J-F, Nassiry M, Mouzdahir A (2007). Free radical oxidation (autoxidation) of α-tocopherol (vitamin E): A potential source of 4,8,12,16-tetramethylheptadecan-4-olide in the environment. Org. Geochem..

[58] Ajoke FL, Haruna K, Ilyas M (2014). Antibacterial activity of 1,2-benzenediccarboxylic acid, dioctyl ester isolated from the ethyl acetate soluble sub-portion of the unripe fruits of *Nauclea latifolia*. Int. J. Pure Appl. Biosci..

[59] Horai H (2010). MassBank: A public repository for sharing mass spectral data for life sciences. J. Mass Spectromet..

[60] Chang C-L, Wu R-T (2011). Quantification of (+)-catechin and (−)-epicatechin in coconut water by LC–MS. Food Chem..

[61] El-Shazly A, Wink M (2014). Diversity of pyrrolizidine alkaloids in the Boraginaceae structures, distribution, and biological properties. Diversity.

[62] Roeder E, Breitmaier E, Birecka H, Frohlicht MW, Badzies-Crombach A (1991). Pyrrolizidine alkaloids of *Heliotropium spathulatum*. Phytochemistry.

[63] Ravi S, Lakshmanan AJ, Herz W (1990). Iso-lycopsamine, a pyrrolizidine alkaloid from *Heliotropium keralense*. Phytochemistry.

[64] Picardo M, Núñez O, Farré M (2020). Suspect and target screening of natural toxins in the Ter River catchment area in NE Spain and prioritisation by their toxicity. Toxins.

[65] Singh A, Bajpai V, Kumar S, Sharma KR, Kumara B (2016). Profiling of gallic and ellagic acid derivatives in different plant parts of *Terminalia arjuna* by HPLC-ESI-QTOF-MS/MS. Nat. Prod. Commun..

[66] Bollinger JG, Rohan G, Sadilek M, Gelb MH (2013). LC/ESI-MS/MS detection of FAS by charge reversal derivatization with more than four orders of magnitude improvement in sensitivity. J. Lipid Res..

[67] Ammar S, Contreras MDM, Belguith-Hadrich O, Bouaziz M, Segura-Carretero A (2015). New insights into the qualitative phenolic profile of *Ficus carica* L. fruits and leaves from Tunisia using ultra-high-performance liquid chromatography coupled to quadrupole-time-of-flight mass spectrometry and their antioxidant activity. RSC Adv..

[68] Tsimogiannis D, Samiotaki M, Panayotou G, Oreopoulou V (2007). Characterization of flavonoid subgroups and hydroxy substitution by HPLC-MS/MS. Molecules.

[69] Farsam H, Yassa N, Sarkhail P, Shafiee A (2000). New pyrrolizidine alkaloids from *Heliotropium crassifolium*. Planta Med..

[70] Shafiei A, Salimi M, Farsam H, Yasa N (2002). Pyrrolizidine alkaloids from *Heliotropium dissitiflorum* Boiss. Daru.

[71] Zalkow L (1979). Pyrrolizidine alkaloids from Middle Eastern plants. J. Nat. Prod..

[72] Constantinidis T, Harvala C, Skaltsounis AL (1993). Pyrrolizidine N-oxide alkaloids of *Heliotropium hirsutissimum*. Phytochemistry.

[73] Lakshmanan AJ, Shanmugasundaram S (1994). Helibractinecine, a pyrrolizidine alkaloid from *Heliotropium bracteatum*. Phytochemistry.

[74] Lakshmanan AJ, Shanmugasundaram S (1995). Heliscabine, a pyrrolizidine ester alkaloid from *Heliotropium scabrum*. Phytochemistry.

[75] Fernández-Fernández R (2010). Simple LC–MS determination of citric and malic acids in fruits and vegetables. Chromatographia.

[76] Al Kadhi O, Melchini A, Mithen R, Saha S (2017). Development of a LC-MS/MS method for the simultaneous detection of tricarboxylic acid cycle intermediates in a range of biological matrices. J. Anal. Methods Chem..

[77] Lin Y-L, Tsai Y-L, Kuo Y-H, Liu Y-H, Shiao M-S (1999). Phenolic compounds from *Tournefortia sarmentosa*. J. Nat. Prod..

[78] Mosaddik A, Forster PI, Waterman PG (2007). Three new 3-benzylbenzofuran-2-one derivatives from *Homalium brachybotrys* (Flacourtiaceae/Salicaceae s. l.). Nat. Prod. Res..

[79] Ricciutelli M (2019). Identification and quantification of new isomers of isopropyl-malic acid in wine by LC-IT and LC-Q-Orbitrap. Food Chem..

[80] Flores AS, de Azevedo Tozzi AMG, Trigo JR (2009). Pyrrolizidine alkaloid profiles in Crotalaria species from Brazil: Chemotaxonomic significance. Biochem. Syst. Ecol..

[81] Lin Y (2015). Qualitative and quantitative analysis of phenolic acids, flavonoids and iridoid glycosides in Yinhua Kanggan tablet by UPLC-QqQ-MS/MS. Molecules.

[82] Fang N, Yu S, Prior RL (2002). LC/MS/MS characterization of phenolic constituents in dried plums. J. Agric. Food Chem..

[83] Stojković D (2021). Extract of Herba *Anthrisci cerefolii*: Chemical profiling and insights into its anti-glioblastoma and antimicrobial mechanism of actions. Pharmaceuticals (Basel, Switzerland).

[84] Barrientos RE (2020). Chemical fingerprinting, isolation and characterization of polyphenol compounds from Heliotropium taltalense (Phil.) IM Johnst and its endothelium-dependent vascular relaxation effect in rat aorta. Molecules.

[85] Simirgiotis MJ (2016). Fast high resolution Orbitrap MS fingerprinting of the resin of *Heliotropium taltalense* Phil. from the Atacama Desert. Ind. Crops Prod..

[86] Dawidowicz AL, Bernacik K, Typek R (2018). Umbelliferone instability during an analysis involving its extraction process. Monatsh. Chem..

[87] Masike K (2017). A metabolomics-guided exploration of the phytochemical constituents of *Vernonia fastigiata* with the aid of pressurized hot water extraction and liquid chromatography-mass spectrometry. Molecules (Basel, Switzerland).

[88] Krzyżanowska-Kowalczyk J, Pecio L, Mołdoch J, Ludwiczuk A, Kowalczyk M (2018). Novel phenolic constituents of *Pulmonaria officinalis* L. LC-MS/MS comparison of spring and autumn metabolite profiles. Molecules.

[89] Brito A, Ramirez JE, Areche C, Sepúlveda B, Simirgiotis MJ (2014). HPLC-UV-MS profiles of phenolic compounds and antioxidant activity of fruits from three citrus species consumed in Northern Chile. Molecules (Basel, Switzerland).

[90] Chen H (2018). Isolation and identification of the anti-oxidant constituents from *Loropetalum chinense* (R. Brown) Oliv. Based on UHPLC–Q-TOF-MS/MS. Molecules.

[91] Singh B, Sahu P, Jain S, Singh S (2002). Antineoplastic and antiviral screening of pyrrolizidine alkaloids from *Heliotropium subulatum*. Pharm. Biol..

[92] Abdel-Sattar E (2009). Antiplasmodial and antitrypanosomal activity of plants from the Kingdom of Saudi Arabia. J. Nat. Med..

[93] Rahman M, Mia M, Shahid I (2011). Pharmacological and phytochemical screen activities of roots of *Heliotropium indicum* Linn. Pharmacologyonline.

[94] Moongkarndi P, Kosem N, Luanratana O, Jongsomboonkusol S, Pongpan N (2004). Antiproliferative activity of Thai medicinal plant extracts on human breast adenocarcinoma cell line. Fitoterapia.

[95] Sadeghi Ekbatan S, Li X-Q, Ghorbani M, Azadi B, Kubow S (2018). Chlorogenic acid and its microbial metabolites exert anti-proliferative effects, S-phase cell-cycle arrest and apoptosis in human colon cancer Caco-2 cells. Int. J. Mol. Sci..

[96] Thangaraj K (2019). Orientin induces G0/G1 cell cycle arrest and mitochondria mediated intrinsic apoptosis in human colorectal carcinoma HT29 cells. Biomolecules.

[97] Arora I, Sharma M, Tollefsbol TO (2019). Combinatorial epigenetics impact of polyphenols and phytochemicals in cancer prevention and therapy. Int. J. Mol. Sci..

[98] Lee HS (2014). Mechanisms underlying apoptosis-inducing effects of Kaempferol in HT-29 human colon cancer cells. Int. J. Mol. Sci..

[99] Molecular Operating Environment (MOE, 2014.0901). https://www.chemcomp.com/Products.htm.

