# Supplementary material for: Heliotropium ramosissimum metabolic profiling, in silico and in vitro evaluation with potent selective cytotoxicity against colorectal carcinoma
Source: Sci Rep. 2022 Jul 22;12:12539. doi: 10.1038/s41598-022-16552-1 (PMC9307647; doi:10.1038/s41598-022-16552-1)
Supplement: Supplementary file 1 — Supplementary Figures. [file 41598_2022_16552_MOESM1_ESM.pdf]

Heliotropium ramosissimum Metabolic Profiling, *In Silico* and *In Vitro* Evaluation with Potent Selective Cytotoxicity Against Colorectal Carcinoma

Marwa A.A.Fayed [a]*, Mohamed E. Abouelela [b], and Mohamed S. Refaey [a]

[a]Department of Pharmacognosy, Faculty of Pharmacy, University of Sadat City, Sadat 32897, Egypt; marwa.fayed@fop.usc.edu.eg, mohamed.said@fop.usc.edu.eg.

[b]Department of Pharmacognosy, Faculty of Pharmacy, Al-Azhar University, Assiut-Branch, Assiut, 71524, Egypt; m_abouelela@azhar.edu.eg

***Corresponding author**: Marwa A.A.Fayed
[a]Department of Pharmacognosy, Faculty of Pharmacy, University of Sadat City, Sadat 32897, Egypt.
E-mail address: marwa.fayed@fop.usc.edu.eg, maafayed@gmail.com
ORCID: https://orcid.org/0000-0001-5609-7436

Table of Contents

No.	Tilte	Page
1	**Fig.S1: Gallic acid standards absorbance and calibration curve,**	2
2	**Fig.S2: Rutin standards absorbance and calibration curve.**	2
3	**Fig. S3: GC-MS chromatogram of *n*-hexane extract of *H. ramosissimum* (Lehm.) DC.**	3
4	**Fig. S4: Structure of the major compounds identified in *n*-hexane fraction of *H. ramosissimum* (Lehm.) DC.**	3
5	**Fig. S5: LC- ESI-MS/MS chromatogram (+ve mode) of the methanolic extract of *H. ramosissimum* (Lehm.) DC.**	4
6	**Fig. S6: Structure of Compounds identified from the LC- ESI-MS/MS (+ ve mode) run of the methanolic extract of *H. ramosissimum* (Lehm.) DC.**	5
7	**Fig. S7: LC- ESI-MS/MS chromatogram (– ve mode) of the methanolic extract of *H. ramosissimum* (Lehm.) DC.**	6
8	**Fig. S8: Structure of the major compounds identified from the LC- ESI-MS/MS (– ve mode) run of the methanolic extract of *H. ramosissimum* (Lehm.) DC.**	7
9	**Fig.S9: IC_{50} of the methanolic extract of the aerial parts using DPPH assay.**	8
10	**Fig.S10: IC_{50} of the methanolic extract of the aerial parts using ORAC assay**	8

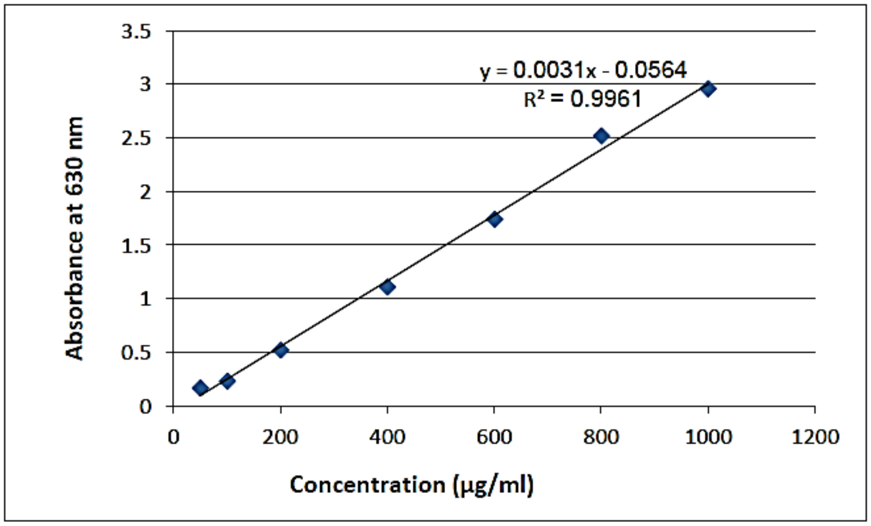

Fig.S1: Gallic acid standards absorbance and calibration curve

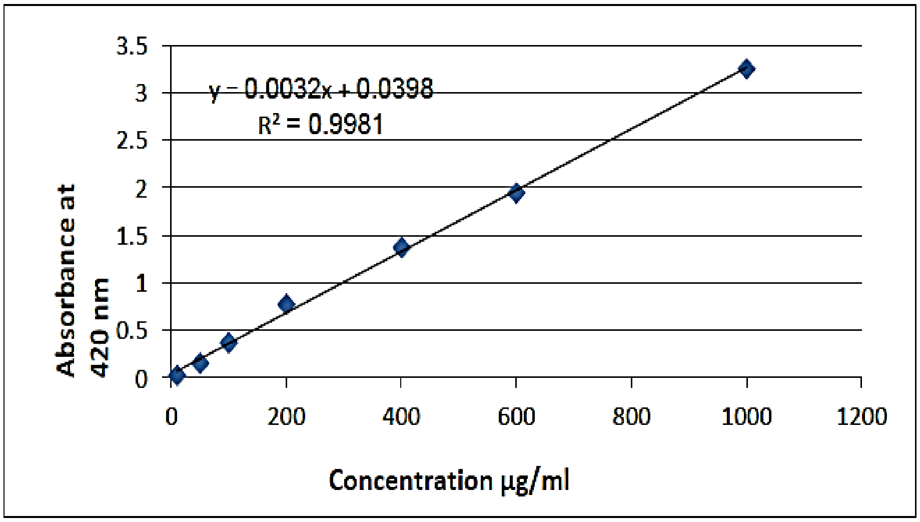

Fig.S2: Rutin standards absorbance and calibration curve

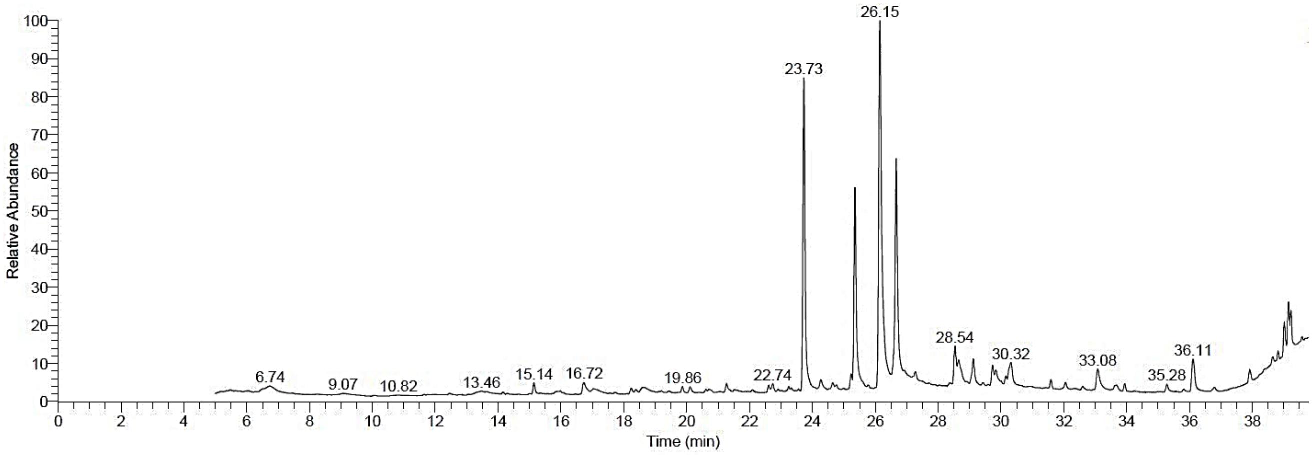

Fig. S3: GC-MS chromatogram of *n*-hexane extract of *H. ramosissimum* (Lehm.) DC.

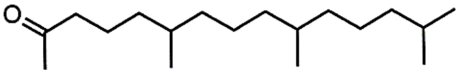

6,10,14-Trimethylpentadecan-2-one

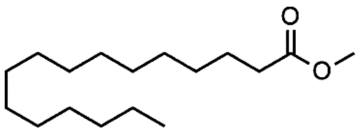

Hexadecanoic acid, methyl ester

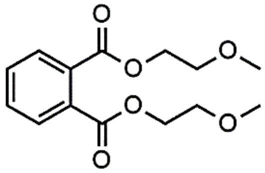

1, 2-Benzenedicarboxylic acid, bis (2-methoxyethyl) ester

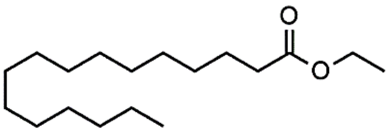

Ethyl hexadecanoate

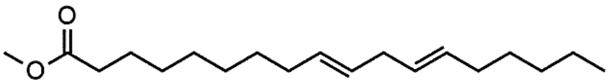

Linolelaidic acid, methyl ester

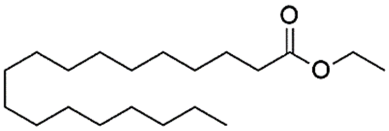

Ethyl octadecanoate

Fig. S4: Structure of the major compounds identified in *n*-hexane fraction of *H. ramosissimum* (Lehm.) DC.

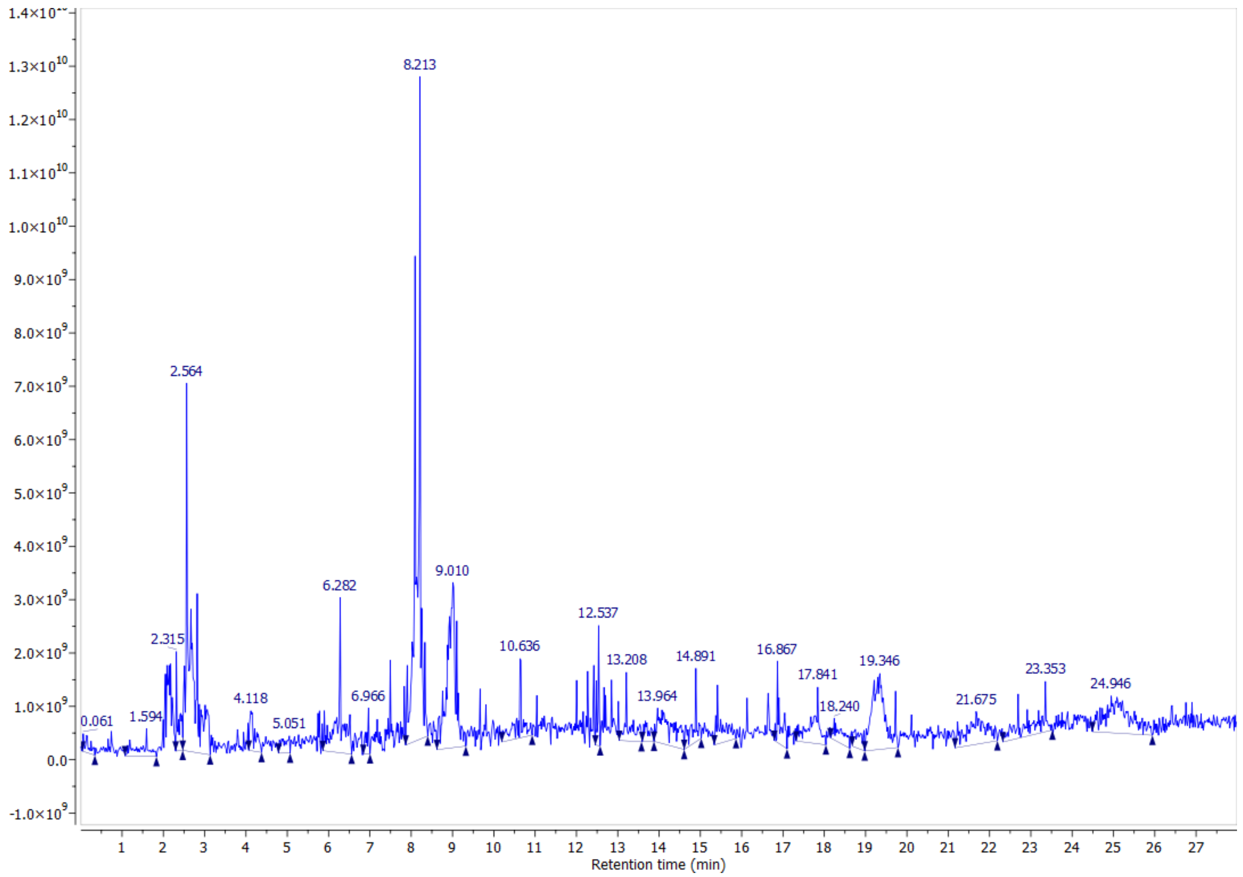

Fig. S5: LC- ESI-MS/MS chromatogram (+ve mode) of the methanolic extract of *H. ramosissimum* (Lehm.) DC.

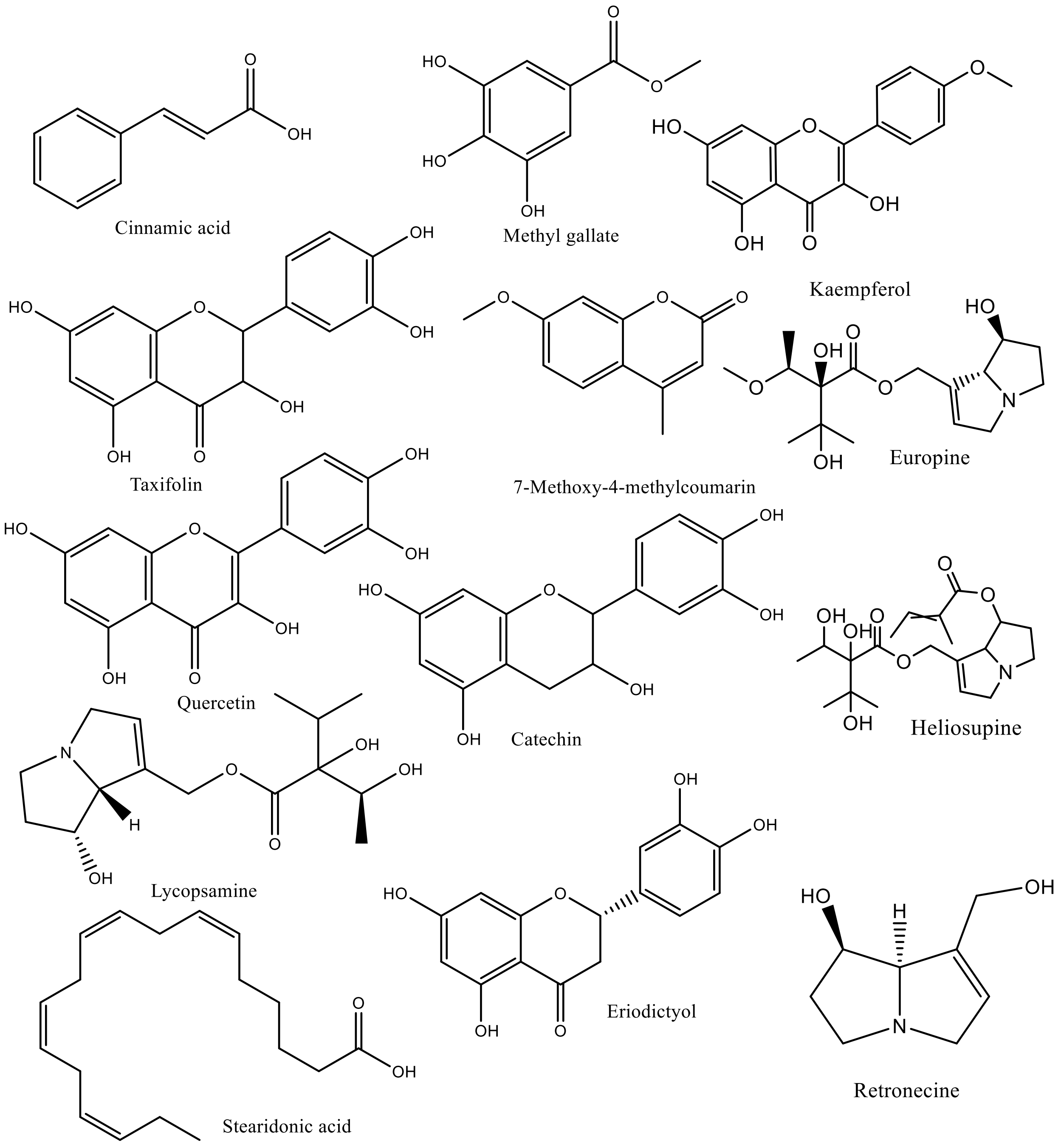

Fig. S6: Structure of Compounds identified from the LC- ESI-MS/MS (+ ve mode) run of the methanolic extract of *H. ramosissimum* (Lehm.) DC.

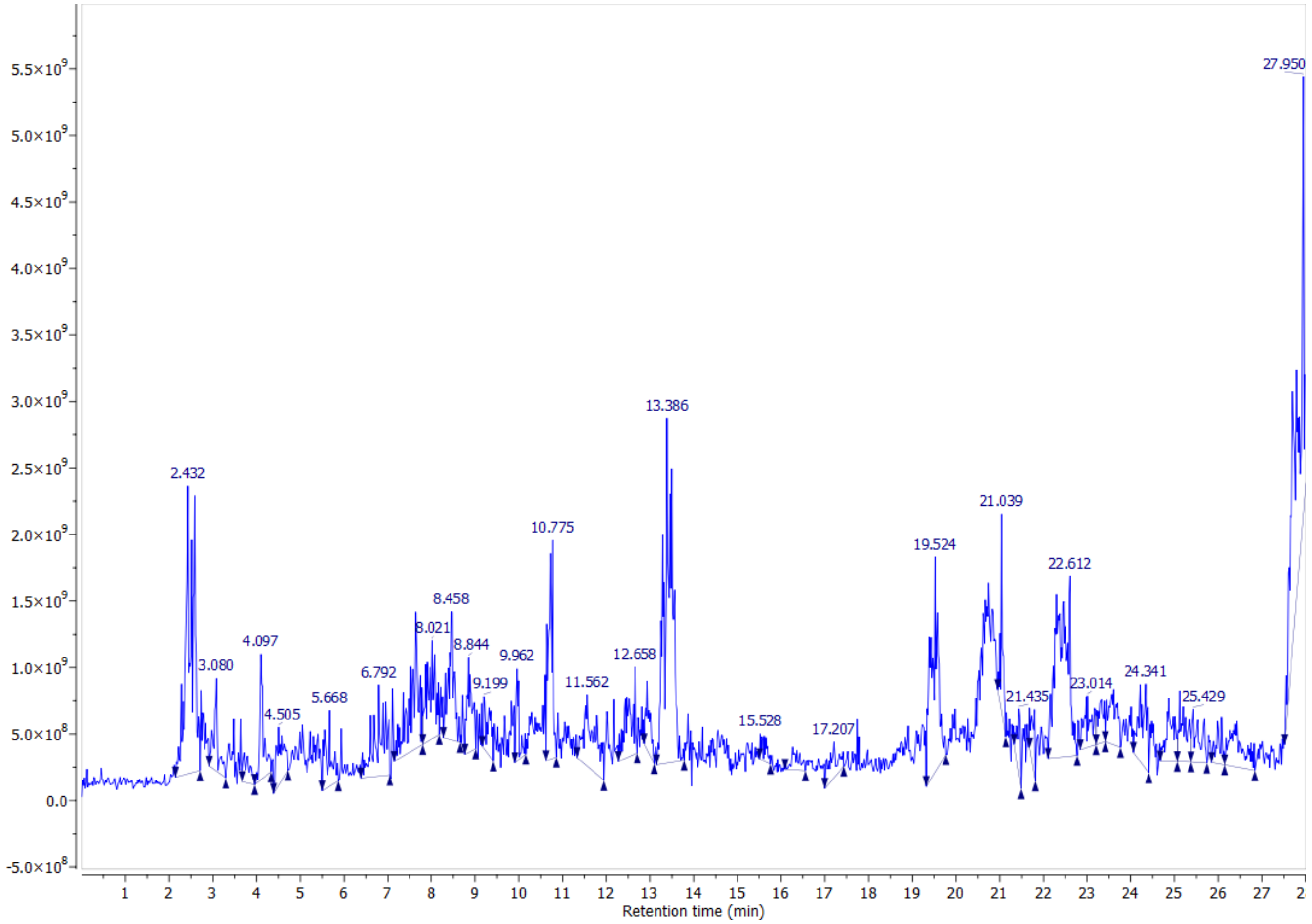

Fig. S7: LC- ESI-MS/MS chromatogram (– ve mode) of the methanolic extract of *H. ramosissimum* (Lehm.) DC.

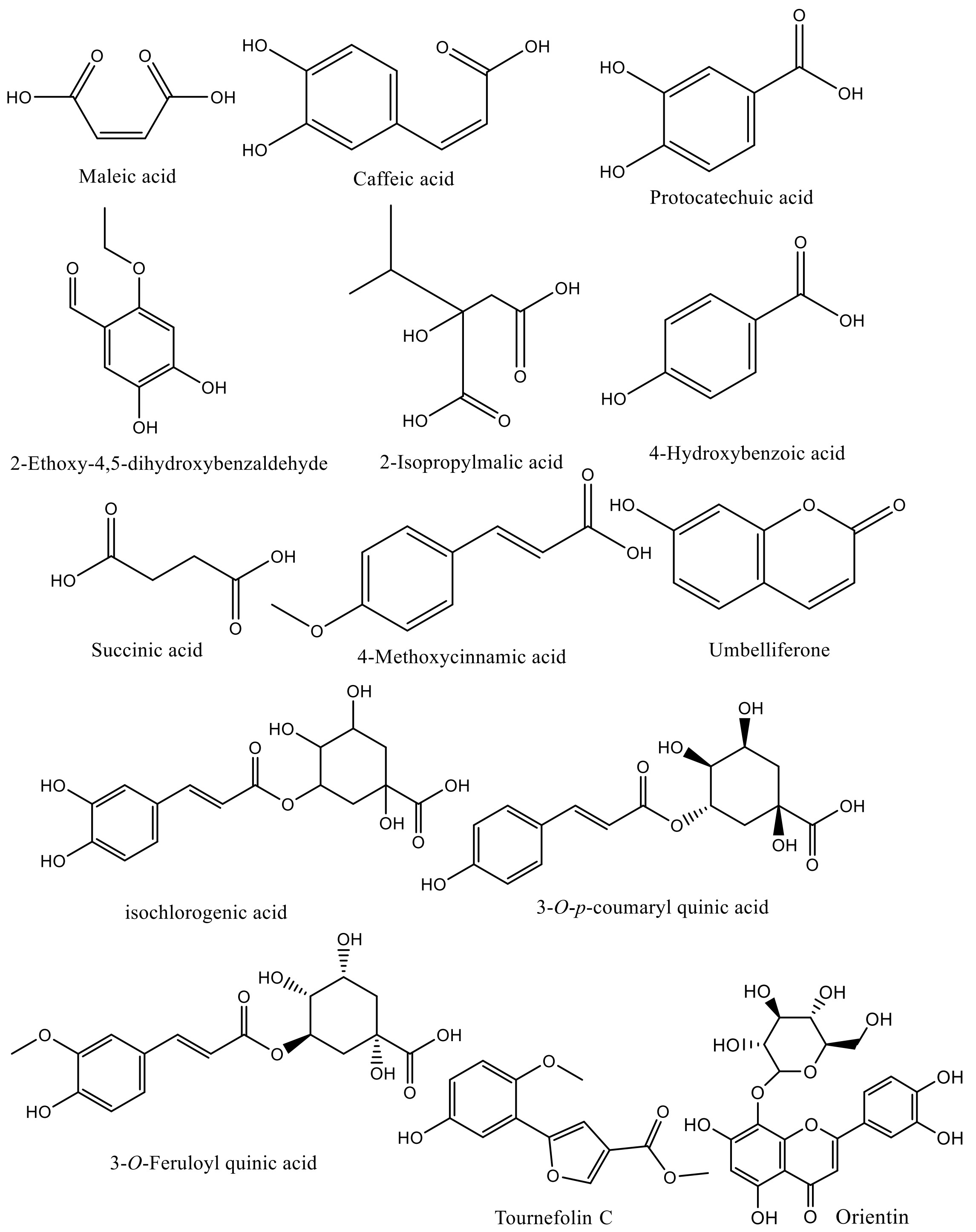

Fig. S8: Structure of the major compounds identified from the LC- ESI-MS/MS (– ve mode) run of the methanolic extract of *H. ramosissimum* (Lehm.) DC.

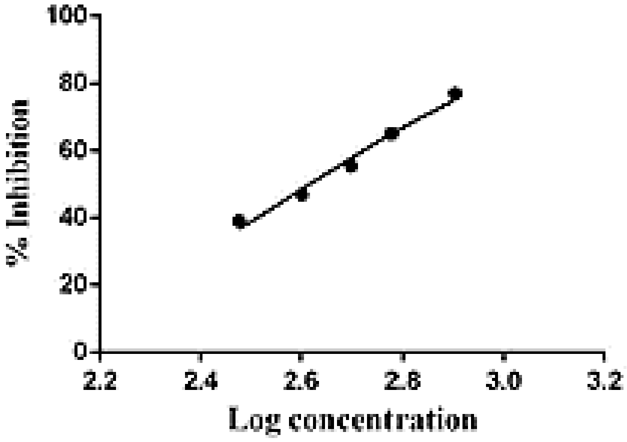

Fig.S9: IC_{50} of the methanolic extract of the aerial parts using DPPH assay.

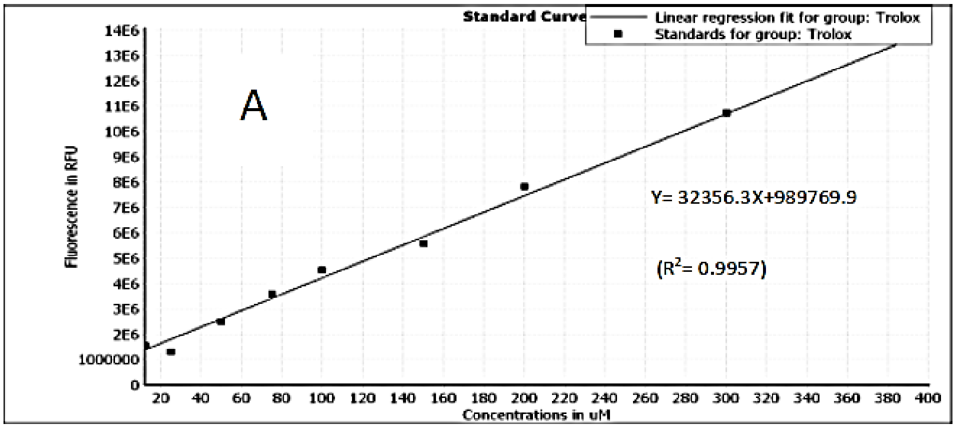

Fig.S10: IC_{50} of the methanolic extract of the aerial parts using ORAC assay
